# Mapping surface-modified titania nanoparticles with implications for activity and facet control

**DOI:** 10.1038/s41467-017-00619-z

**Published:** 2017-09-22

**Authors:** Yung-Kang Peng, Yichen Hu, Hung-Lung Chou, Yingyi Fu, Ivo F. Teixeira, Li Zhang, Heyong He, Shik Chi Edman Tsang

**Affiliations:** 1 The Wolfson Catalysis Centre, Department of Chemistry, University of Oxford, Oxford, OX1 3QR UK; 2 Department of Chemistry and Shanghai Key Laboratory of Molecular Catalysis and Innovative Materials, Fudan University, Shanghai, 200433 People’s Republic of China; 3 Graduate Institute of Applied Science and Technology, National Taiwan University of Science and Technology, Taipei, 10617 Taiwan

## Abstract

The use of surface-directing species and surface additives to alter nanoparticle morphology and physicochemical properties of particular exposed facets has recently been attracting significant attention. However, challenges in their chemical analysis, sometimes at trace levels, and understanding their roles to elucidate surface structure–activity relationships in optical (solar cells) or (photo)catalytic performance and their removal are significant issues that remain to be solved. Here, we show a detailed analysis of TiO_2_ facets promoted with surface species (OH, O, SO_4_, F) with and without post-treatments by ^31^P adsorbate nuclear magnetic resonance, supported by a range of other characterization tools. We demonstrate that quantitative evaluations of the electronic and structural effects imposed by these surface additives and their removal mechanisms can be obtained, which may lead to the rational control of active TiO_2_ (001) and (101) facets for a range of applications.

## Abstract (web summary)

Metal oxide nanocrystals can be grown with different facets exposed to give variations in reactivity, but the chemical state of these surfaces is not clear. Here, the authors make use of a phosphine probe molecule allowing the differences in surface chemistry to be mapped by NMR spectroscopy.

## Introduction

Surface features and distributions on metal oxides such as oxygen vacancies (V_o_), cations, anions and hydroxyl groups have been shown to play a decisive role in various important applications such as optics, electronic devices and heterogeneous catalysis^1–5^. In particular, for nano-sized particles, the domination of these surface features has been demonstrated to lead to the particles exhibiting different physical/chemical properties compared with the bulk. Given that each facet possesses a distinctive intrinsic energy, it is understandable that both the concentration and chemical state of the surface features differ from facet to facet. Taking ZnO nanocrystallite as an example, the surface V_o_ were shown to be present on the oxygen-terminated (002) facet^6^, and were recently quantified by Peng et al.^7^ Also, the chemical properties of surface zinc ions have been shown to be different on (100) and (002) facets, indicating the Lewis acidity of ZnO NP can be modulated by the morphology control^7^. These considerations have provided additional variables in tailoring the morphology of nanoparticles (NPs) with preferentially exposed facets during the past decades. Unfortunately, facets with high reactivity are unstable and tend to diminish rapidly to minimize surface energy during the crystal growth^8^. Accordingly, a structure-directing species (SDS) or surfactant is usually employed in the shape control of NPs, which renders them in metastable and high-energy forms. The SDS can subtly modify the chemical state of the surface features (especially metal cations) and provide a kinetic growth control for the formation of shaped NPs^1^. As the growth rate of a crystal facet depends on the surface energy, different morphologies of NP could thus be prepared by using various SDS^8, 9^.

The tailoring of anatase TiO_2_ crystals with the termination of specific facets has received great interest for many years. However, most synthetic TiO_2_ crystals are covered with thermodynamically more stable (101) facets rather than the reactive (001) facets^10, 11^. The breakthrough in controlling the synthesis of crystals with a high percentage of reactive high-energy (001) sites was not made until 2008^12^. According to that literature, the relative stability of these two facets can be reversed using a small quantity of fluorine on the (001) surface during the particle synthesis. Since then, the controlled synthesis of TiO_2_ nanocrystals with preferentially exposed (001) facets has been shown to display promising properties in photocatalysis^13–24^, methanol conversion^25^, solar cells^26–29^ and lithium batteries^30, 31^, etc. Generally the removal of SDS, with fluorine in this case, after the controlled nanoparticle synthesis is required to avoid interference with the desired surface properties. It can be removed by a simple post-treatment step such as calcination at 600 °C^12, 14–16, 19, 21, 23, 26, 27, 30^ or ion exchange with aqueous NaOH^13, 15, 17–19, 24^ before the application to obtain a so-called ‘clean surface’. However, by adopting different removal methods, diverse results have been obtained in the literature in various applications (e.g., photocatalysis cases summarized in Table 1), which have led to different interpretations and frequent disagreements among researchers^13–31^. For example, the NaOH-treated (001) facet (i.e., Na-(001)) showed higher activity in the photocatalytic degradation of methyl orange dye than the F-capped (001) facet (i.e., F-(001))^13^, while a reverse result was obtained for the same reaction by another research group^15^ (Table 1). Also, both NaOH and calcination treatments were reported to increase the facet activity of (101) in photocatalytic hydrogen evolution^16, 17^, while the NaOH treatment was concluded to lower the facet activity of (001) in this reaction by others^17, 18^. These different observations can arise from the surface reconstruction of the unstable (001) surface upon its removal during calcination^32, 33^ or traces of residual SDS that remain on the surface affecting the chemical state of exposed titanium (vide infra). So far, no clear rationalization or guidance for the selection of appropriate post-treatment methods has been achieved. In addition, the SDS modification on the TiO_2_ surface has been demonstrated to efficiently modulate the surface Ti chemical state. For example, the post-modification of PO_4_^3-^/SO_4_^2-^ on the TiO_2_ surface can provide extra Brønsted acid sites and also increase Lewis acidity for enhanced sugar conversion^34, 35^/photocatalysis^36, 37^. The chelating of surface COOH-containing dye molecule^38^and electrolyte additive^39^, 4-*t*-butylpyridine, have been found to remarkably improve the solar cell performance due to the modification of the TiO_2_ conduction band (unfilled d-band of Ti^4+^) for efficient electron transfer in dye-sensitized solar cells.

**Table 1:** Facet-dependent photocatalytic reaction of anatase TiO_2_ NPs prepared using fluorine as SDS

Ref.	Post-treatment	Photocatalytic reaction	Activity	Proposed mechanism
13	NaOH wash	Degradation of MO	Na-(001)>F-(001)	Reactive (001) facet
14	Calcination	Hydroxyl radical production	Cal-(001)>F-(001)	Density of unsaturated Ti
15	NaOH wash/calcination	Degradation of MO and MB	MO: F-(001)>Na-(001)–Cal-(001)MB: Na-(001)>Cal-(001)>F-(001)	Selective adsorption
16	Calcination	Hydroxyl radical production/H_2_ evolution from water	Cal-(010)>Cal-(101)>Cal-(001) >F-(010)–F-(101)–F-(001)	Density of unsaturated Ti/B and structures
17	NaOH wash	H_2_ evolution from water	Na-(101)>F-(101)>F-(001)>Na-(001)	Density of unsaturated Ti
18	NaOH wash	H_2_ evolution from water	F-(001)>Na-(001)–TiO_2_	Surface fluorination
19	NaOH wash/calcination	Degradation of acetone	F-TiO_2_>Cal-TiO_2_–Na-TiO_2_	Synergetic effect of (001) facet and surface fluorination
20	–	Degradation of acetaldehyde	F-TiO_2_>TiO_2_	Surface fluorination
23	Calcination	CO_2_ reduction	(001): oxidation (101): reduction	Synergetic effect of (001) and (101) facets
24	NaOH& HF wash	Degradation of MB	HF-Na-TiO_2_>Na-TiO_2_>TiO_2_	Synergetic effect of two facets and surface defects

Facet-controlled fluorine-capped anatase TiO_2_ NPs treated with various post-treatments (*Na* NaOH wash, *Cal* calcination) and their corresponding activities/mechanisms. F-(001) represents as-prepared F-capped TiO_2_ NP preferentially exposes (001) facet; Na/Cal-(001) represents F-(001) post-treated with NaOH wash/calcination for the removal of surface fluorine

Given the importance of the facet-dependent properties of TiO_2_ nanoparticles and their chemical modifications for a wide range of applications, to the best of our knowledge there is still no detailed work on the mapping of surface features of functionalized TiO_2_ with different morphologies. Herein, using anatase TiO_2_ samples of different morphologies, we successfully monitor the subtle changes of the Ti chemical state induced by a small quantity of surface adsorbates (i.e., fluorine, sulfate, OH) on various facets using ^31^P magic angle spinning (MAS) nuclear magnetic resonance (NMR) in combination with trimethylphosphine (TMP) as a surface probe. This is based on the fact that the nucleophilic probe TMP molecule can form a stable adduct with the exposed Ti cation (Lewis acid (LA)) of the TiO_2_ surface, and the formation of a surface TMP-Ti complex can be realized via coordination of the P atom to the surface Ti cation centre. According to ^31^P chemical shift (δ^31^P) of the corresponding surface TMP-Ti complexes, it is demonstrated the surface Ti cations on various facets with different Lewis acidities, surface energies and steric arrangements can be carefully mapped, differentiated and quantitatively analysed. It is shown that some typical post-treatments (i.e., calcination or NaOH wash) to remove the fluorine (as SDS) can also lead to different surface Ti chemical states. According to our catalytic testing, extra caution must be exercised for even trivial surface treatments as a huge difference in activity can be obtained. This technique (i.e., using TMP as a surface probe) is thus demonstrated as a powerful tool in combination with conventional surface techniques adopted for TiO_2_ NPs such as X-ray photoelectron spectroscopy (XPS), electron paramagnetic resonance (EPR) and Raman spectroscopy to offer comprehensive information of surface features on various functionalized facets to guide surface treatments/modifications of TiO_2_ NP for various applications.

## Results

### TiO_2_ nanocrystals and their surface chemical states

**Fig. 1:**
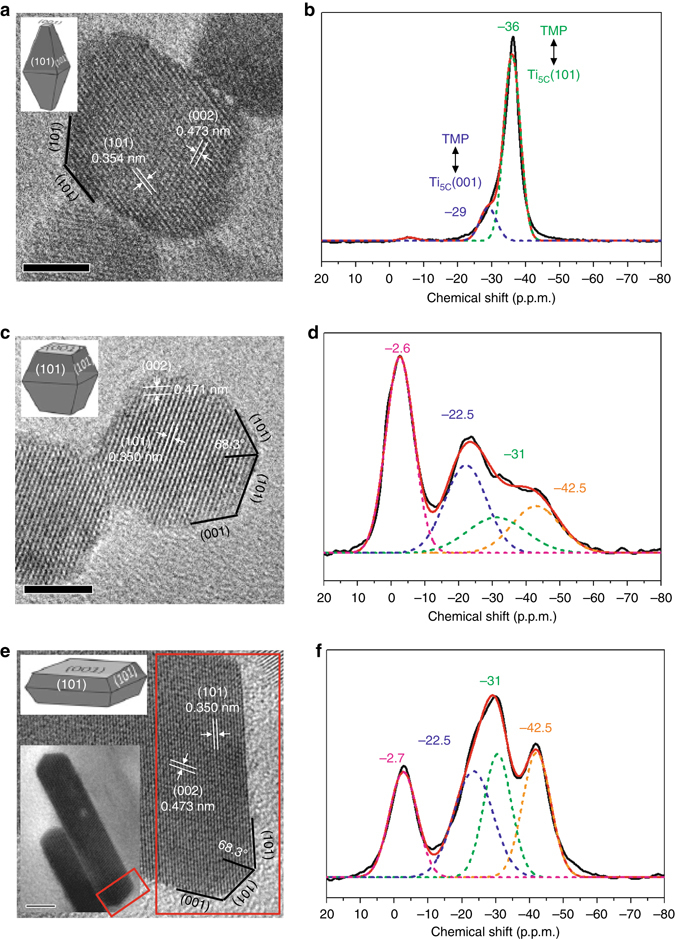
TEM images of as-prepared TiO_2_ samples and corresponding ^31^P MAS NMR spectra of TMP-adsorbed samples. TEM images of as-prepared **a** powder (PD), **c** F-(101), **e** F-(001) and **b**, **d**, **f** their corresponding spectra of TMP-adsorbed samples. *Green* represents TMP of surface 5-coordinate Ti^4+^ on (101) facet (i.e., Ti_5C_(101)), *blue* represents TMP of 5-coordinate Ti^4+^ on (001) facet (i.e., Ti_5C_(001)), *orange* represents TMP of surface Ti^4+^ containing OH and F and *pink* represents TMP on Brønsted acid site (TiO(H)-Ti). The F-(001) sample clearly show a higher coverage of (001) facet reflected by the largest corresponding ^31^P chemical shift of *blue peak* (cf., *green peak*); while F-(101) with higher coverage of (101) facet and PD with the highest coverage of (101) facet both show a dominant *green peak*. See NMR spectrum deconvolution section in Experimental section for details. The *scale bar* in all images is 5 nm

Samples prepared with 0, 2 and 6 mL of 50 wt% concentration of hydrogen fluoride (HF) reveal different morphologies and are labelled as powder (PD) (Fig. 1a), F-(101) (Fig. 1c) and F-(001) (Fig. 1e). All samples exhibited lattice fringes with d-spacings around 0.47 and 0.35 nm, in accordance with the [101] and [002] crystallographic planar directions of anatase TiO_2_, respectively. According to Wulff construction, the proportion of exposed (101) and (001) facets in as-prepared samples can be calculated (Supplementary Fig. 1). The powder sample was characterized with ~90% (101) facet, matching well with the thermodynamically stable (101) facet in anatase TiO_2_ predicted by Wulff construction (~94%)^40^. These particles gave an average face length of 3.8 ± 0.5 nm (Fig. 1a and Supplementary Fig. 2a). According to the literature, when the concentration of HF is increased, it can increase the particle face length due to preferential adsorption of F to slow down its growth on this facet and hence the percentage of exposed (001) facet increases (Supplementary Table 1). Indeed, when 2 mL HF was used in our case, an average face length of ca. 6.6 ± 1.0 nm was achieved (Fig. 1c and Supplementary Fig. 2b) with a substantially higher proportion of (001) facet (Supplementary Table 1). By using 6 mL HF, the elongated face length of ca. 41.0 ± 10.5 nm with a thickness of ca. 6.2 ± 0.9 nm nanosheet-like F-(001) particles were obtained (Fig. 1e and Supplementary Fig. 2c). The percentages of exposed (101) and (001) facets of F-(001) were estimated to be 24.6 and 75.4%, respectively (Supplementary Table 1).

Figure 1b, d, f shows the corresponding ^31^P MAS NMR spectra of TMP-adsorbed powder, F-(101) and F-(001) samples. This technique clearly gives distinctive fingerprint-like NMR patterns for TiO_2_ samples with various surface exposures/treatments. In general, the chemical shift of ^31^P (δ^31^P) in a range of −2 to −5 p.p.m. has been well attributed to the formation of a TMPH^+^ ionic complex formed when a TMP molecule is protonated (Brønsted acid site), while the δ^31^P of adsorbed TMP spans over a wide range (−20 to −58 p.p.m.) when interacting with surface acidic site (LA site) with various Lewis acidity (the formation of TMP-LA) (Supplementary Note 1 and Supplementary Fig. 3)^41^. Accordingly, a stronger interaction of the surface acidic site with the basic TMP molecule is expected to give a more positive δ^31^P shift in the NMR spectrum, and the magnitude of this shift should depend on the adsorption energy/geometry on a particular facet and the TMP molecule^7^.

In order to compare these NMR peaks over the samples with different proportions of facets, ^31^P MAS NMR peaks in the spectra of TMP-adsorbed TiO_2_ have been carefully deconvoluted. The powder sample shown in Fig. 1b reveals no significant formation of TMPH^+^ complex between −2 and −5 p.p.m. but TMP-LA complex with a main peak at −36 ± 1 p.p.m. and a small shoulder at lower chemical shift (−29 ± 1 p.p.m.) are clearly evident. The error of ±1 p.p.m. includes variations in facet dimension, defect concentration and measurement inaccuracy in the heterogeneous solid system. Notice that similar results in chemical shift values have been shown by Deng and co-workers^37^ over their titanium oxide. The major peak at −36 p.p.m. and the shoulder at −29 p.p.m. with the integrated area ratios of 84.8 and 15.2% respectively can be attributed to the interaction between TMP and surface five-coordinate Lewis acids: Ti^4+^on the (101) facet as Ti_5C_(101) and Ti^4+^ on (001) as Ti_5C_(001), respectively. This high proportion value of (101) facet in our powder sample matches with our earlier estimation using Wulff construction (89.8%, Supplementary Table 1).

**Fig. 2:**
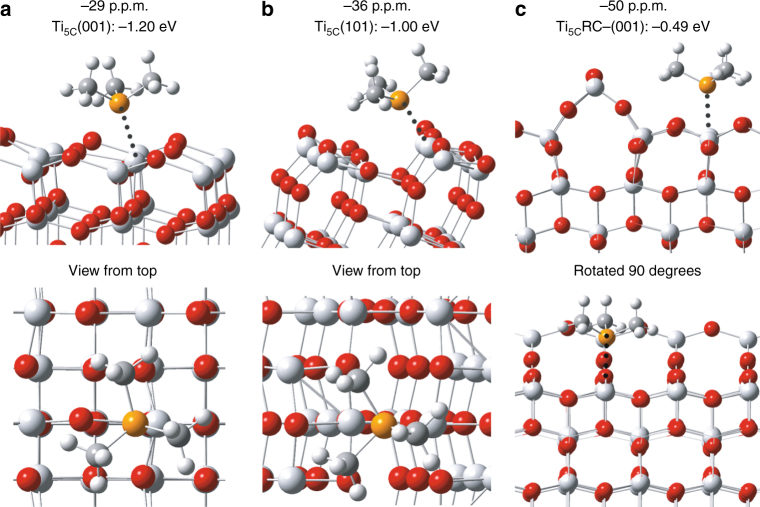
Calculated results of molecular interaction. Schematic illustrations of molecular interaction and DFT calculated adsorption energy (E_ad_) between TMP and various TiO_2_ facets. **a** Ti_5C_(001), **b** Ti_5C_(101) and **c** Ti_5C_RC-(001). RC-(001) represents reconstructed (1 × 4) (001) facet (Ti: *light grey*; O: *red*; P: *orange*; C: *grey*; H: *white*)

It has been observed that the adsorption energy of TMP molecule on the Lewis acid site shows a strong correlation with its NMR chemical shift value^7, 41–43^. As shown in Fig. 2, our calculated adsorption energies of TMP on Ti_5C_(001), Ti_5C_(101) and Ti_5C_ RC(reconstructed)(001) are obtained according to computational models, respectively. Indeed, it is found that the calculated adsorption energy displays an excellent linear relationship with the experimental chemical shift value obtained (Supplementary Table 2 and Supplementary Fig. 4). Thus, this NMR technique is capable of differentiating facets of decreasing energy: (001)>(101)>RC(001) by their chemical shift values (−29, −36 and −50 p.p.m.), respectively. The relatively higher Brønsted acid signal of around −3 p.p.m. of F-(001) sample shown in Fig. 1f as compared to that of F-(101) sample shown in Fig. 1d again clearly indicates the preferential exposure of the (001) facet due to the increase in F concentration used.

On the other hand, some immediate questions remain to be answered from Fig. 1b, d, f. First, why did the resolved (101) peak of chemical shift −31 ± 1 p.p.m. and (001) peak of −22.5 ± 1 p.p.m. from the samples prepared with HF (i.e., F-(101) and F-(001)) show a consistent and significant 5 to 7 p.p.m. downshift from corresponding facets of −36 and −29 p.p.m. in the powder sample (no fluorine contamination), respectively? What is the nature of the peak with a chemical shift of −42.5 ± 1 p.p.m., which was not seen over the PD sample? Why did the samples with HF treatment (i.e., F-(101) and F-(001)) show both Brønsted acid and Lewis acid signals but the as-prepared powder sample (PD) possessed mainly Lewis acid signal?

**Fig. 3:**
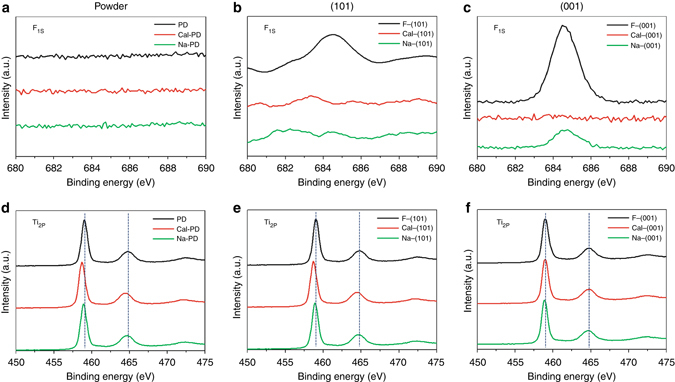
XPS study of TiO_2_ samples. XPS F_1S_ spectra of as-prepared **a** PD, **b** F-(101) and **c** F-(001) TiO_2_ samples with different treatments (calcination and NaOH wash). **d** to **f** are the corresponding XPS Ti_2P_ spectra

XPS, a technique to monitor element(s) on material surface, was used to analyse the surface compositions of these samples. As shown in Fig. 3a–c, the surface F on powder, F-(101) and F-(001) samples can be monitored by XPS as previously reported^12^. It is noted that F is apparently retained on the facets (101) and (001) when the samples were prepared with F (Fig. 3b, c) but the powder sample without previous exposure to F shows the total absence of F_1S_ signal (Fig. 3a). Despite the fact that XPS has been regarded as a surface-sensitive technique to atomic chemical states, it fails to give any chemical shift of Ti_2P_ in the presence of fluorine (Fig. 3d–f). It is clear that the long electrons escape depth, greater than the outermost atomic layer (few atoms depth) of the sample, and this renders XPS unsuitable for surface studies in this context (detection limit ~0.1% atom) and the monitoring of core electrons makes the binding energy of Ti also less sensitive to electronic effects from neighbouring F element(s). Auger electron spectroscopy is known to provide higher spatial resolution and the energy of ejected Auger electrons is more sensitive to the chemical environment compared to the core-level signals in XPS. However, as shown in Supplementary Fig. 5, no peak shift was also observed for five Ti LMM Auger signals (marked by *dashed blue line*). This indicates the change of chemical state of the outermost Ti atom is still averaged out during the collection of Auger electrons from the electron escape depth. In contrast, the ^31^P NMR is much more surface sensitive to the electronic change imposed by this SDS.

The 5 to 7 p.p.m. downshift in values of the F-(101) and F-(001) samples from the corresponding facets of −36 p.p.m. Ti_5C_(101) and −29 p.p.m. Ti_5C_(001) in the powder sample to −31 ± 1 p.p.m. and −22.5 ± 1 p.p.m., respectively, are likely due to the presence of the electronic withdrawing effect of fluorine exerted to the Ti^4+^ on these two facets. Note that a surface-bound ligand molecule generates an electric dipole, while this intrinsic ligand dipole depends on its chemical structure and binding mode. For Lewis-basic ligands, the interfacial dipole points from the ligand toward the metal (L^*δ*–^→M^*δ*+^) and the largest ligand-induced downward shift of electronic energy levels is observed for halide ion ligands^1^. This electronic effect can be supported by our DFT calculation on a fluorinated (001) model. As shown in Supplementary Fig. 6, the calculated adsorption energy between TMP and Ti_5C_ on the clean (001) facet (−1.20 eV, Fig. 2) is greatly increased to −1.76 eV when the surrounding Ti_5C_ is fluorinated. According to the correlation plot between experimental δ^31^P and calculated adsorption energy in Supplementary Fig. 4, this can explain the downfield shift of F-(001) observed within experimental error. It is thought that a similar downfield shift for the F-(101) facet occurs upon the fluorination of surface Ti_5C_ at close proximity. The corresponding atomic ratios (Ti, O and F) of TiO_2_ samples by XPS are summarized in Supplementary Table 3. The apparent lower Ti:O values in the presence of F clearly suggest the formation of V_o_ due to the strong electronic withdrawing effect of the surface fluorine groups. EPR (Supplementary Note 2, Supplementary Fig. 7 and Supplementary Tables 4 and 5) and Raman (Supplementary Note 3 and Supplementary Fig. 8) could also be used to indicate the electronic and structural changes of facets imposed by this surface impurity. Thus, such unstable (001) facets with F-containing surface oxygen vacancies could be hydrolysed (introduction of the OH functional group) during the treatment to account for the −42.5 ± 1 p.p.m. peak observed (see later experiments). In addition, the evolution of Brønsted acidity over the fluorine-contaminated facets can be rationalized by the surface hydrogen bonding stabilization of protons by the fluorine. As a result from this study, one has to be very careful about the detainment of surface additive on a particular facet which can subtly affect its electronic, structural and geometric properties (acidity), and hence the evaluation of facet-dependent properties. Thus, post treatment to remove SDS before the study of facet-dependent properties is crucially important.

**Fig. 4:**
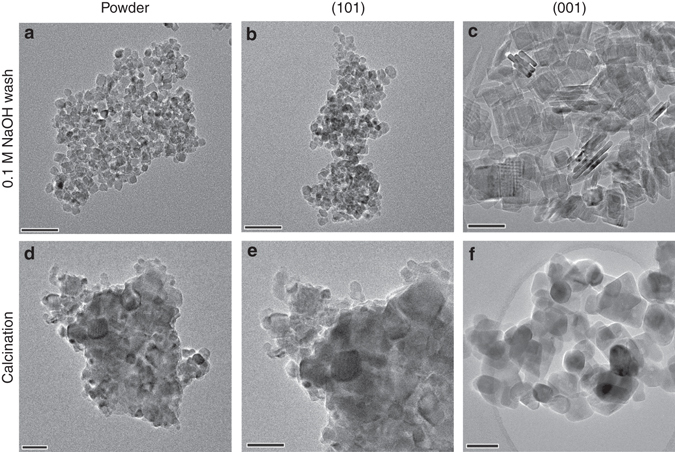
Morphology of TiO_2_ samples after different treatments. TEM images of TiO_2_ samples after **a**–**c** NaOH wash: **a** Na-PD, **b** Na-(101) and **c** Na-(001) and **d**–**f** after calcination treatment: **d** Cal-PD, **e** Cal-(101) and **f** Cal-(001). The *scale bar* in all images is 50 nm

As stated, a NaOH wash^13, 15, 17–19, 24^ and calcination treatment^12, 14–16, 19, 21, 23, 26, 27, 30^ of F-treated samples at elevated temperature are the two commonly used methods in literature to remove surface F after the controlling of TiO_2_ particle morphology. The apparent remaining F signal on Na-(001) sample (cf. Cal-(001)) after NaOH (0.1 M) treatment shown in Fig. 3c clearly indicates that the F removal was not complete presumably due to the higher F affinity for this energetic surface. In contrast, there is no F found in all calcined samples by the XPS (Fig. 3). However, this does not necessary suggest that calcination is a good method since high energetic facets may easily reconstruct to more stable surface at high temperature^32, 33^. Moreover, as evidenced by TEM images (Fig. 4), calcination treatment causes severe particle aggregation for all three as-prepared PD, F-(101) and F-(001) while NaOH treatment does not lead to any observable aggregation and change in morphology. This result can be further supported by their corresponding XRD patterns (Supplementary Fig. 9). These two post-treatments according to our surface analysis could generate artefacts when facet-dependent properties are studied.

### NaOH wash to remove surface fluorine

**Fig. 5:**
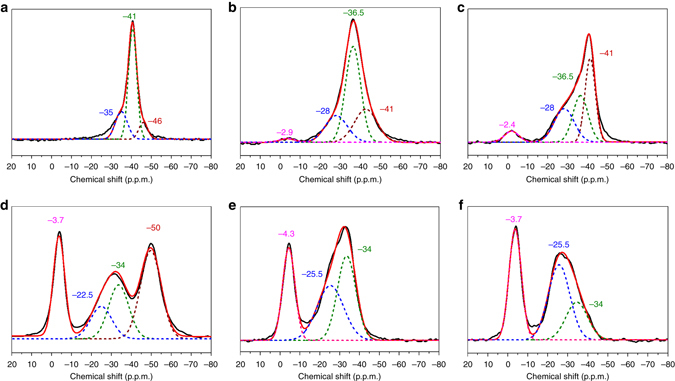
NMR results of NaOH-washed TiO_2_ samples and their following sulfation treatment. The ^31^P MAS NMR spectral deconvolution of TMP-adsorbed TiO_2_ samples treated with 0.1 M NaOH: **a** Na-PD, **b** Na-(101) and **c** Na-(001) and their following sulfation treatment: **d** S-Na-PD, **e** S-Na-(101) and **f** S-Na-(001)

As 0.1 M NaOH has been widely adopted in literature to remove surface fluorine^13, 15, 17–19, 24^, ^31^P MAS NMR spectra of TMP-adsorbed Na-PD, Na-(101) and Na-(001) were also collected and are deconvoluted in Fig. 5. In order to reduce the influence of NaOH on the measured chemical shift values, the samples after the NaOH treatment were repeatedly washed with deionized (DI) water. Notice that no significant residual NaOH was retained on these surfaces (Supplementary Note 4 and Supplementary Fig. 10). For samples prepared without HF (i.e., PD) (Fig. 5a), the NaOH treatment after rinsing with DI water clearly reduces the Lewis acidity of both Ti_5C_(001) and Ti_5C_(101) as δ^31^P shifts from −29 p.p.m. to −35 ± 1 p.p.m. for Ti_5C_(001) and from −36 p.p.m. to −41 ± 1 p.p.m. for Ti_5C_(101), respectively, for the surface mono-hydroxylation (cf. as-prepared PD, Fig. 1b). A shoulder appears at higher field (~ 46 ± 1 p.p.m.) which is attributed to the formation of multi-hydroxylation Ti_5c_O_5-*x*_(OH)_*x*_ with *x* > 2 during the extensive hydrolysis by the NaOH treatment at high concentration (Supplementary Fig. 11)^44, 45^. However, for F-treated samples (i.e., F-(101) or F-(001)), a different Lewis acid distribution is obtained after the same treatment (Fig. 5b, c). The conversion of residual F-Ti to HO-Ti on high energetic facets, particularly on (001), by OH- exchange in the NaOH wash can give a very different electron density of Ti_5C_ atoms compared to surface Ti without the F. The surface chemical functionalization by OH shifts the δ^31^P of TMP-adsorbed F-Ti_5C_(001)/F-Ti_5C_(101) from −22.5 ± 1/−31 ± 1 p.p.m. (Fig. 1d, f) to −28 ± 1/−36.5 ± 1 p.p.m. (Fig. 5b, c). In addition, the typical Brønsted acid signals (the formation of TMPH^+^) between −2 and −5 p.p.m. clearly indicate that there is a residue F left on both Na-(101) and Na-(001) sample surfaces (Fig. 5b, c). On the other hand, the total F-depleted surfaces of −35 ± 1 p.p.m. for Ti_5C_(001) and −41 ± 1 p.p.m. for Ti_5C_(101) are expected to re-emerge since NaOH is capable of removing some surface F. The former peak (−35 p.p.m.) is unfortunately mixed with the OH-shifted F-Ti_5C_(001) peak (−36.5 p.p.m.) but the prominent at −41 ± 1 p.p.m. can be attributed to the reformation of Ti_5C_(101) (Supplementary Fig. 11) during its surface hydrolysis by the NaOH treatment.

### Post-calcination to remove surface fluorine

Recently, the heat-induced aggregation of sheet-like anatase TiO_2_ has been shown to occur preferentially along the [001] crystallographic direction, driven by the minimization of surface energy^46, 47^. According to Yang et al.^46^, surface Ti-F groups are totally hydrolysed during calcination in humid air at high temperature (endothermic reaction) (Supplementary Fig. 12). In this case, the exposed surfaces of sheet-like anatase TiO_2_ are also mainly covered by -OH groups. As the (001) facet has a higher surface energy than the (101) facet, the (001) interfaces between adjacent nanosheets would be eliminated through the condensation of Ti-OH groups (formation of Ti-O-Ti linkages) at high temperature and therefore the (101) facet becomes dominant (Supplementary Fig. 12). According to our XPS (Fig. 3), TEM (Fig. 4)/high-resolution TEM (Supplementary Fig. 13) and XRD (Supplementary Fig. 9) data, the post calcination is effective to remove F from (001) and (101) surfaces, which can clearly induce aggregation.

The ^31^P MAS NMR spectra of TMP-adsorbed Cal-PD and Cal-(001) were deconvoluted and are shown in Supplementary Fig. 14a, b. It is noted that calcination in humid air pushes the δ^31^P of Ti_5C_(001)/Ti_5C_(101) of PD from −29/−36 to −35 ± 1/−41 ± 1 p.p.m. due to the surface mono-hydroxylation as mentioned previously (cf. as-prepared PD, Fig. 1b). Interestingly, Supplementary Fig. 14a shows a new but characteristic signal of Cal-PD at −50 ± 1 nm. According to our DFT calculation (i.e., −50 p.p.m., Fig. 2), this is due to the adsorption of TMP on Ti_5C_ on (1 × 4) the reconstructed (001) facet (Ti_5C_RC-(001)). Thus, this peak can be used as a diagnostic peak to indicate reconstruction/aggregation induced by calcination. However, for F-(001), the same chemical shifts for Ti_5C_(001)/Ti_5C_(101) (i.e., −35 ± 1/−41 ± 1 p.p.m.) indicative of the mono-hydroxylation of the surfaces with no residual F can be obtained by calcination (Supplementary Fig. 14b) but not for the NaOH wash at room temperature, where retention of F on Ti_5C_(001)/F-Ti_5C_(101) is evident (−28 ± 1/−36.5 ± 1 p.p.m., see Fig. 5c). The large signal of Cal-(001) at −45 ± 1 p.p.m. (Supplementary Fig. 14b) can be assigned to the metastable (1 × 3) and (1 × 5) (001) facets originating from the dominant high energy but unstable (1 × 1) (001) facet before their further transformation to the more stable reconstructed (1 × 4) structure (at −50 p.p.m.)^33^. By comparing the area of deconvoluted peaks of Cal-(001), we are able to show an ∼80% of (001) facet that had been reconstructed during the calcination.

### Surface sulfation

As stated, surface functionalization could severely alter facet properties but this issue has not been much addressed in previous literature. The sulfation of TiO_2_ facets was therefore investigated after the calcination or NaOH wash with a prolonged period of time to ensure no F remained on the surfaces. The ^31^P MAS NMR result for calcined samples after sulfation treatment (i.e., S-Cal-PD and S-Cal-(001)) are shown separately in Supplementary Fig. 14c, d. As calcination treatment causes severe aggregation and unavoidable facet reconstruction, we herein focus on the sulfation of NaOH-washed samples. For F-(101) and F-(001) samples, Brønsted acid (Ti-O(H)-Ti) sites removed by NaOH wash can clearly be reintroduced by a sulfation step with a large and distinctive peak around −4 p.p.m. (Fig. 5e, f). It is interesting to note that two new and characteristic strong Lewis acid peaks due to TMP adsorption on sulfated surfaces, namely −25.5 ± 1 and −34 ± 1 p.p.m., are introduced. It is postulated these two new Lewis acid sites are arisen from the Ti^4+^ modified directly by SO_4_ at high surface coverage during the extensive sulfation but further verifications are required. Their strengths (peak shifts) are sensitive to the particular facets and their relative concentrations (peak areas) also depend on the relative proportions of (101) and (001) in the samples. For instance, S-Na-(101) with the preferentially exposed (101) facet shows a stronger signal at −34 ± 1 p.p.m. (Fig. 5e), while for S-Na-(001) with the preferentially exposed (001) facet gives a more dominant signal at −25.5 ± 1 p.p.m. (Fig. 5f). For the PD samples after the extensive hydrolysis during treatments, surface multi-hydroxylation is expected to give a peak at −50 ± 1 p.p.m., as well as the two Lewis acid peaks of −25.5 ± 1and −34 ± 1 p.p.m. (Fig. 5d).

### Qualitative and quantitative analysis

**Fig. 6:**
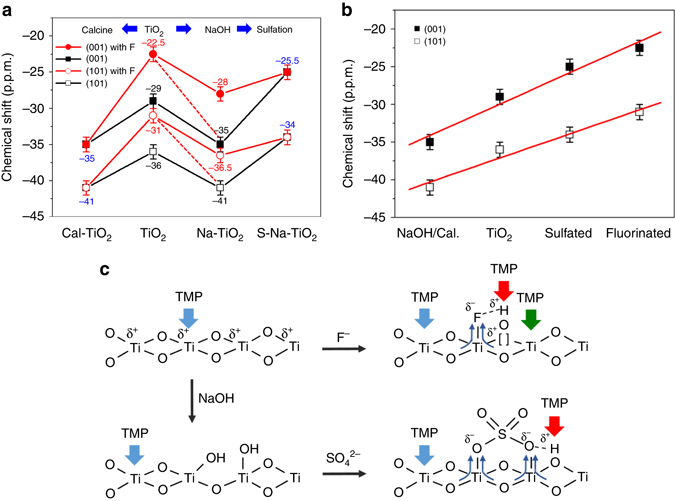
Interaction between TMP and surface features on TiO_2_ facet with various treatments/modifications. **a** The δ^31^P of TMP-adsorbed Ti_5C_ on (001)/(101) facets (from PD) and on F-modified (001)/(101) facets (from F-(001) and F-(101)) with different treatments/modifications. **b** The summary of δ^31^P of TMP-adsorbed Ti_5C_ on (001)/(101) facets with different treatments (calcination/NaOH wash) and modification (sulfate/fluorine). The *error bar* in **a**, **b** is ±1 p.p.m. **c** Illustration of interaction between TMP and surface features on TiO_2_ facet with various treatments/modifications

Figure 6 and Table 2 summarize qualitative (chemical shift) and quantitative (peak area) information of deconvoluted peaks in TMP-adsorbed TiO_2_^31^P MAS NMR spectra under various treatments. First, Fig. 6a shows that the adsorption of TMP on Lewis acid centres of the higher energy (001) facet gives lower chemical shift value (−29 ± 1 p.p.m.) than that of the more stable (101) facet (−36 ± 1 p.p.m.) in our samples. F-treatment of the (001) and (101) facets can clearly enhance the Lewis acidity, shifting them to −22.5 ± 1 and −31 ± 1 p.p.m., respectively. This surface group with the highest electron withdrawing ability can also generate oxygen vacancies, and hence activating the surfaces substantially. However, it is clear from our study that calcination in humid air at elevated temperature can indeed remove the surface F groups by introducing hydroxylation to the surfaces. Replacing F, with a strong withdrawing propensity, by much weaker surface OH group will render the upshifts to −35 ± 1 p.p.m. for the (001) facet and −41 ± 1 p.p.m. for the (101) facet due to mono-hydroxylation as mentioned previously (multi-hydroxylation can further upshift the δ^31^P of the adsorbed TMP). However, the high coverage of OH particularly on (001) facet at elevated temperature during calcination can concomitantly lead to severe aggregation. For a prolonged period of time, this can lead to stepwise reconstruction of (1 × 1) (001) facets to the more stable reconstructed (1 × 4) structure (at −50 p.p.m.). On the other hand, the replacement of surface F with OH using a NaOH wash at room temperature can also achieve the F removal but retain the integrity of desired facets and prevent significant aggregation and reconstruction, giving the same shift values of −35 ± 1 p.p.m. for the (001) facet and −41 ± 1 p.p.m. for the (101) facet. However, our TMP-adsorption technique supported by other characterizations (XPS, EPR, Raman) show clearly that a NaOH wash at different times and concentrations at room temperature may not be sufficient to remove all surface F from the TiO_2_. If F is retained together with the mono-hydroxylated OH, it can give two new δ^31^P values (different Lewis acid strengths) of TMP-adsorbed F-Ti_5c_(001)/F-Ti_5c_(101) of −28 ± 1/−36.5 ± 1 p.p.m. (Fig. 6a). In addition, two strong Brønsted acid strengths (the formation of TMPH^+^) at −2.9 and −2.4 p.p.m. (Fig. 5b, c) are also achieved due to the proton stabilization by residual F left on both Na-(101) and Na-(001) sample surfaces. Upon extended sulfation after the samples are calcined (Supplementary Fig. 14c, d) or NaOH (Fig. 5e, f) washed to replace all F with SO_4_, weaker Brønsted acid strengths between −3.7 and −4.7 p.p.m. with protons influenced by neighbour SO_4_ are obtained. In terms of Lewis acid alteration, −25.5 ± 1 p.p.m. from sulfation of (001) and −34 ± 1 p.p.m. of (101) facets can be tuned. Figure 6b shows the highly sensitive δ^31^P value towards increasing electron withdrawing ability to the surface Ti^4+^ from OH<-O-<SO_4_<F which can provide fine-tuning of Lewis acid and Brønsted acid sites on TiO_2_ facets by these surface additives. Figure 6c summarizes the anticipated molecular interactions between TMP and surface features on TiO_2_ with various treatments/modifications. In addition, the TMP-assisted NMR technique displays quantitative assessments on the surface speciation (Table 2). The total adsorbed TMP over different samples also shows strong correlations with the total BET (Brunauer–Emmett–Teller) surface areas of the samples (BET of S-Na-PD = 107.0 m^2^/g, S-Na-(101) = 77.3 m^2^/g and S-Na-(001) = 67.4 m^2^/g (Supplementary Table 6), which is proportional to their total TMP/g (both Brønsted acid and Lewis acid from Table 2) uptakes of 512.6, 286.7 and 261.4 μmol/g, respectively). Typically, this technique shows a better surface sensitivity; for example, 5.8 μmol/g Brønsted acid stabilized by F on Na-(101) is detectable by the NMR (Table 2) but no corresponding F signal is observed by XPS (Fig. 3b). It is particularly noted that XPS fails to reveal the change in Ti_2P_ electronic state with or without fluorine (Fig. 3d–f) but ^31^P MAS NMR can lead to their detailed assessments (Table 2).

**Table 2:** Qualitative and quantitative results of all surface features on TiO_2_ facet with various treatments and modifications

Sample	Brønsted acid (−2 to −5 p.p.m.)		Lewis acid sites (−20 to −58 p.p.m.)						
	p.p.m.	Total TMP^a^	Ti_5c_(001) (p.p.m.)	TMP^a^	Ti_5c_(101) (p.p.m.)	TMP^a^	Others (p.p.m.)	TMP^a^	Total TMP^a^
F-(001)	−2.6	89.4	−22.5	53.2	−31	32.0	−42.5	33.1	118.3
Cal-(001)	–	–	−35	21.9	−41	32.9	−45/−50	61.6/28.6	145.0
Na-(001)	−2.4	30.9	−28	135.1	−36.5	149.1	−41	181.6	465.8
S-Cal-(001)	−4.7	70.7	–	–	–	–	–	–	–
S-Na-(001)	−3.7	91.4	−25.5	116.4	−34	53.6	–	–	170.0
F-(101)	−2.7	50.1	−22.5	72.4	−31	57.1	−42.5	60.9	190.4
Na-(101)	−2.9	5.8	−28	96.0	−36.5	222.6	−41	117.8	436.4
S-Na-(101)	−4.3	83.6	−25.5	97.5	−34	105.6	–	–	203.1
PD	–	–	−29	106.8	−36	605.3	–	–	712.1
Cal-PD	–	–	−35	12.7	−41	66.2	−50	5.9	84.8
Na-PD	–	–	−35	143.3	−41	393.9	−46	59.7	596.9
S-Cal-PD	−3.9	85.4	–	–	–	–	–	–	–
S-Na-PD	−3.7	128.7	−25.5	75.6	−34	117.1	−50	191.2	383.9

Summarizes the qualitative (chemical shift) and quantitative (peak area) of each deconvoluted peak in the region of Brønsted acid site (−2 to −5 p.p.m.) and Lewis acid site (−20 to −58 p.p.m.). The concentration of adsorbed TMP on each site was calculated according to corresponding peak area (^a^Adsorbed TMP molecules in μmol/g)

### Catalytic testing

**Fig. 7:**
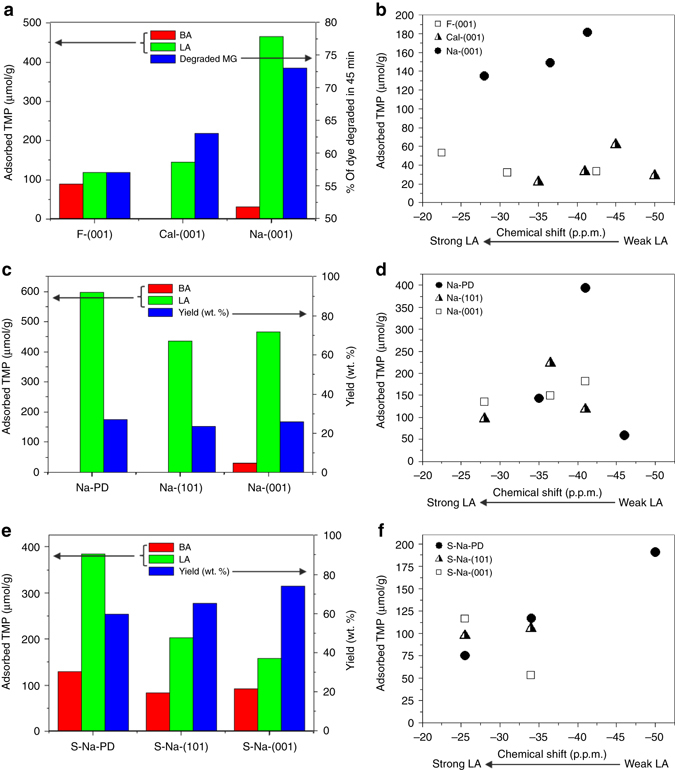
TiO_2_ catalytic activity and the distribution of corresponding active sites. Comparison of Brønsted acid (BA) and Lewis acid (LA) for **a** photocatalytic decomposition rate of malachite green (MG) dye in 45 min over F-(001) samples with various treatments. Pechmann condensation activity over **c** NaOH-treated samples and **d** further sulfated samples. **b**, **d**, **f** are corresponding concentrations of Lewis acid sites of tested samples with different strengths

It is well accepted that photocatalytic activity is highly dependent on particular facets of transition metal oxide crystals. Pioneered by Han et al.^13^ and Yang et al.^14^, using sheet-like TiO_2_ with a higher percentage of reactive (001) facets was proven to exhibit higher photocatalytic activity in aqueous medium (cf., P25). Both of the above reports also showed that the photocatalytic efficiency of the TiO_2_ nanosheets can be further improved after removing surface fluorine either by calcination^12, 14^ or NaOH wash^13^. However, the changes in the quantity and quality of active sites on this (001) facet upon the above treatments were still unable to offer detailed analysis and assessment due to the lack of appropriate characterization techniques^12–31^. To illustrate the importance of acquiring the delicate surface electronic and structural changes imposed by a particular facet and surface chemical modifier, the influence of various treatments to TiO_2_ surface features were monitored (Table 2). The photocatalytic decomposition rate of malachite green (MG) upon F-(001), Cal-(001) and Na-(001) in static solution was therefore studied to reassess the surface structure–photocatalytic activity relationships^13–24^. Corresponding results are shown in Fig. 7a. For samples with the preferential exposure of (001) facet, the NaOH-washed F-(001) sample (i.e., Na-(001)) shows the highest photo-decomposition efficiency while the sample without any treatment (i.e., F-(001)) shows the lowest efficiency (activity: Na-(001)>Cal-(001)>F-(001)). This result indicates that the key factors are neither crystallinity nor surface area. This is because F-(001) possesses similar crystallinity (Supplementary Fig. 9)/surface area (Supplementary Table 6) to Na-(001) and has a higher surface area than Cal-(001) but shows the lowest activity. The role of surface defect such as V_o_ is also proved to be minor as both XPS Ti to O ratio (Supplementary Table 3) and quantitative EPR signal at g~2.0 (Supplementary Table 4) suggest the V_o_ concentration is in a reverse order: F-(001)>Cal-(001)>Na-(001). There is also no significant change in their measured bandgap values (Supplementary Fig. 15), which is commonly reported to be one of the important factors in photocatalysis^16^. According to our NMR results, the possibility of Brønsted acid playing a key role is also ruled out as the Cal-(001) with no detectable Brønsted acid exhibits a higher percentage of MG degradation rate than F-(001), which has the highest Brønsted acid concentration in solution. The acid strength of Lewis acid also does not seem to play the key role in this reaction either as F-(001) with the strongest Lewis acid site (at ~−22 p.p.m., Fig. 7b) exhibits the lowest activity. In this case, the total Lewis acid concentration (465.8 μmol/g of Na-(001)>145 μmol/g of Cal-(001)>118.3 μmol/g of F-(001)) appears to be the primary cause for this activity (Fig. 7a) as it is proportional to the rate and yield of photodecomposition of the MG molecules. As the fluorine binds strongly to the surface Ti atom (blocking the Lewis acid site) on (001) facet, it is not surprising that its removal by NaOH wash in the Na-(001) sample with similar particle size (cf., F-(001)) has quadrupled the Lewis acid surface concentration. Even though severe aggregation has taken place over the calcined Cal-(001) sample, it still possesses a higher Lewis acid concentration than that of F-(001). A similar conclusion can also be drawn for samples with preferentially exposed (101) facets (i.e., PD, see Supplementary Note 5 and Supplementary Fig. 16 also for commercial P25). This indicates that the surface feature and surface area are the key factors for the observed difference in the decomposition rates. This result can be supported by the observation of our previous study which found that the small degree of adsorption of dye molecules due to limited Lewis acid sites was the rate-determining step for photocatalytic reaction in the Nb_2_O_5_ case in static solution^48^. However, the photocatalytic reaction carried out in the flowing air seems to depend on Lewis acid strength more than the total Lewis acid concentration. Xiang et al.^19^ reported that F-TiO_2_ gives better activity than treated TiO_2_ (i.e., Cal-TiO_2_ and Na-TiO_2_) for the photocatalytic degradation of acetone in flowing air. They suggested the strong electron withdrawing ability of the surface Ti–F groups reduces the recombination of photo-generated electrons and holes and enhances the photocatalytic activity. However, according to our result, we believe the adsorption dynamic of acetone in flowing air is very different from that of dye in static water and can only be efficiently adsorbed from the gas phase by the stronger Lewis acid site of F-TiO_2_ (e.g., −22 p.p.m., Fig. 7b) before the photodecomposition. Thus, the stronger nature of Lewis acid site of F-TiO_2_ renders higher activity than that of the treated TiO_2_ (i.e., Cal-TiO_2_ and Na-TiO_2_) in the gas phase degradation of acetone in air.

To demonstrate the importance of assessing the electronic and structural states on facets both qualitatively and quantitatively in heterogeneous catalysis, the catalytic performances of Na-PD, Na-(101), Na-(001) and corresponding sulfation samples (i.e., S-Na-PD, S-Na-(101) and S-Na-(001)) for the catalytic Pechmann condensation reaction using phloroglucinol and ethyl acetoacetate as starting reagents were carried out and the results are shown in Fig. 7c, e. In view of the Pechmann reaction, the condensation of phenol and β-keto ester proceeds through transesterification followed by intramolecular hydroalkylation and dehydration^49^. As shown in Fig. 7c, the yield of 5,7-dihydroxy-4-methyl coumarin of Na-PD (27.13 wt.%) ≥ Na-(001) (25.86 wt.%) = Na-(101) (23.53 wt.%) in solution shows a slight correlation with total Lewis acid concentration but both Lewis acid strength (Fig. 7d) and the trace Brønsted acid of Na-(001) show almost no significant influence (total concentration of Lewis acid is more important than its strength in solution catalysis as previously discussed). However, the yield is almost tripled when using corresponding sulfation samples in the order of S-Na-(001) (73.87 wt.%)>S-Na-(101) (65.16 wt.%)>S-Na-PD (59.68 wt.%) (Fig. 7e). This huge increase in yield for all the three samples could be easily mistakenly attributed to the introduction of weak Brønsted acid by sulfate modification at the expense of Lewis acid sites (see Fig. 7c, e). However, S-Na-PD with the highest total concentrations in both Brønsted acid and Lewis acid shows the lowest yield among the three samples and similar Brønsted acid strength is actually obtained for all the three samples. Thus, Brønsted acid does not seem to be the main site for this catalysed reaction. Notice that the sulfate modification of S-Na-PD, S-Na-(101) and S-Na-(001) also concomitantly introduces a new Lewis acid site of much greater strength than unmodified samples (Fig. 7f). The strength of this newly generated Lewis acid site at ~−25 p.p.m. (Ti^4+^ modified directly by the neighbours SO_4_ and OH, see Fig. 6c) is found to be comparable to sulfated/BF_3_-modified metal oxides (SO_4_^2−^/ZrO_2_^50^, BF_3_/Al_2_O_3_^51^) which is characteristic of super Lewis acidity. This Lewis acid concentration is clearly found to be in the order of S-Na-(001)>S-Na-(101)>S-Na-PD (Fig. 7f) in accordance with the order of their product yield, which undoubtedly suggests that the specific concentration of this new-generated super acid site is responsible for catalysing this reaction in solution accordingly.

## Discussion

**Fig. 8:**
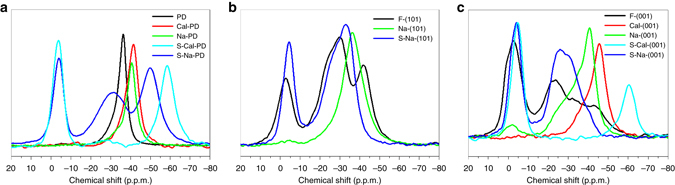
Superposition of ^31^P NMR spectra of TMP-adsorbed TiO_2_ samples. Summary of the electronic effect (chemical shift) imposed by different adsorbates during sequential treatments/modifications on **a** PD, **b** F-(101) and **c** F-(001) with various exposed facets

The nanometre dimensions and intrinsic heterogeneity of NPs (each particle typically exposes several facets with different patterns of surface atoms) makes the experimental study of surfaces challenging. Traditional surface tools such as XPS, EPR and Raman spectroscopy have been widely employed to bridge facet-controlled NPs and their corresponding facet-dependent performances. However, as demonstrated in this study (using TiO_2_), these techniques (XPS: Fig. 3; EPR: Supplementary Fig. 7; Raman: Supplementary Fig. 8) can only provide limited information on the chemical state of surface cations and their distribution among facets, causing difficulty in explaining the corresponding facet-dependent results. Moreover, various processing steps and post-treatment techniques (e.g., NaOH/calcination treatment here) used during the preparation of NPs from group to group further complicate surface environments and always lead to different interpretations and disagreements among researchers. Herein, qualitative and quantitative information on both the chemical state and distribution of surface cations among facets promoted with various groups have been well resolved by the use of an NMR-active probe (Fig. 8). We are also able to monitor the electronic effect imposed by different adsorbates during sequential treatments/modifications to the NP surface cation (Supplementary Figs. 17 and 18). It is noted that the employment of a NMR chemical probe to aid the characterization of the acidity of zeolite was introduced firstly by Lunsford and colleagues (1984)^52^ and is utilized for various solid acid catalysts nowadays^53^. However, to the best of our knowledge there is no report regarding its potential use in differentiating the adsorbate-dependent chemical state of cations on various crystal facets, which can be crucial for filling the gap between the model catalysts used in surface science and the real catalysts found in practical applications.

In conclusion, it is clear from this study that the type and quantity of surface Ti electronic states and structures are highly sensitive to the intrinsic nature and amount of particular facets, SDS and additives used as well as treatment methods adopted (calcination or NaOH wash), etc. They play decisive role(s) for a wide range of TiO_2_ applications, especially optical and thermal catalysis, which are closely associated with the coordination environment and the chemical state of surface titanium atoms at the catalyst surface. Hence, we believe that this study gives insights to resolve the current debates and confusions in some facet-dependent properties in literature, should more comprehensive surface characterization including ^31^P adsorbate-assisted MAS NMR be invoked.

## Methods

### Synthesis of F-(001), F-(101) and powder TiO_2_ sample

A total of 5.0 mL of Ti(OC_4_H_9_)_4_ was mixed with a certain amount of hydrofluoric acid (40–48 wt.%) in a Teflon-lined autoclave with a capacity of 40 mL and subsequently heated to 180 °C at a ramp rate of 2 °C/min. The temperature was kept at 180 °C for 24 h^13^. After hydrothermal reaction, the white precipitate was collected, washed with ethanol and distilled water three times and then dried in an oven at 80 °C overnight. Then, 0.6 and 0.2 mL of hydrofluoric acid was added for F-(001) and F-(101), respectively, while, instead of hydrofluoric acid, 0.6 mL of H_2_O was employed for powder (PD) sample.

### NaOH removal of surface fluorine

The NaOH wash was employed in this study according to previous literature^13^. Then, 1.0 g of as-prepared TiO_2_ sample was treated in 50.0 mL of 0.1 M NaOH solution under magnetic stirring for 10 h (0.05 and 0.5 M NaOH were also adopted for comparison). The solid sample was then washed with distilled water several times until neutral. After centrifugation, the solid was dried at 80 °C overnight. TiO_2_ samples (i.e., PD, F-(101) and F-(001)) obtained after a 0.1 M NaOH wash were denoted as Na-PD, Na-(101) and Na-(001).

### Calcination removal of surface fluorine

The calcination treatment was also carried out according to a previous report^12^. Then, 1.0 g of as-prepared TiO_2_ sample was calcined at 600 °C for 90 min (ramping rate: 5 °C/min). We denoted TiO_2_ samples after receiving this treatment as Cal-PD, Cal-(101) and Cal-(001).

### Sulfation of TiO_2_

To obtain the sulfated TiO_2_, ammonium sulfate ((NH_4_)_2_SO_4_) was utilized as the precursor of sulfate in the catalyst preparation^54^. For example, 1.0 g of Na-PD/Cal-PD sample pretreated over a prolonged period of time was added into 10.0 mL of 1 mol/L (NH_4_)_2_SO_4_ solution to stir for 8 h and then collected by centrifugation. The solid was then calcined at 450 °C for 4 h. We denoted Na-PD and Cal-PD sample after sulfation as S-Na-PD and S-Cal-PD. The same treatments were also employed to the other two morphologies.

### XPS, EPR and Raman measurement

XPS measurements were recorded on a Thermo Scientific K-Alfa XPS instrument equipped with micro-focused monochromated Al X-ray source. The source was operated at 12 keV and a 400 μm spot size was used. The analyzer operated at the analyzer energy (CAE) of 200 eV for survey scans and 50 eV for detailed scans. Charge neutralization was applied using a combined low energy/ion flood source. The data acquisition and analysis were conducted with CasaXPS (Casa software Ltd). The peak position was referenced to C1s peak of the carbon tape at 285.00 eV. The EPR spectra were obtained by X-band CW EPR spectrometer (Bruker EMX) and the signal intensity vs. electron spin number was obtained from the double integral of the spectrum. Raman spectra were measured with a Raman Microscope (Renishaw) with a laser excitation wavelength of 532 nm. Exposure time of 10 s and 8 number scans were adopted for each measurement.

### TMP-adsorbed sample preparation

Approximately 150 mg of TiO_2_ sample was placed in a home-made glass tube and activated at 150 °C for 2 h under vacuum (10^−1^ Pa) (Supplementary Fig. 19). After cool down to room temperature, 300 μmol/catalyst g (calculated by the pressure and volume of isolated system) of TMP was then introduced. There was a wait of approximately 10 min for the equilibrium between TMP and catalyst surface to be reached. Extra TMP molecules were removed by vacuum system. These steps were repeated three times to ensure full adsorption of TMP on catalyst surface. The sample tube was then flame sealed for storage and transferred to Bruker 4 mm ZrO_2_ rotor with a Kel-F endcap in a glove box under nitrogen atmosphere before NMR measurement (see Supplementary Note 6 for more details).

### NMR measurement

The solid-state MAS NMR experiments were carried out using a Bruker Avance III 400WB spectrometer at room temperature. MAS speed of all our samples was 12 kHz. The high-power decoupling was used for the quantitative ^31^P analysis. Considering the long relaxation time of ^31^P nuclei in NMR experiment, we used 30° pulse with the width of 1.20 μs, 15 s delay time. The radiofrequency for decoupling was 59 kHz. The spectral width was 400 p.p.m., from 200 to −200 p.p.m. The number of scanning was 800. The ^31^P chemical shifts were reported relative to 85% aqueous solution of H_3_PO_4_, with NH_4_H_2_PO_4_ as a secondary standard (0.81 p.p.m.). The quantitative analysis of adsorbed TMP molecules was calculated according to the calibration line established by running standard samples with various adsorbed TMP concentration (see Supplementary Note 7, Supplementary Figs. 20–22 and Supplementary Table 7 for more details).

### NMR spectrum deconvolution

All raw TMP NMR spectra were deconvoluted using the software ‘peakfit v4.12’. We employed ‘gauss area’ and ensured all results with *R* ^2^ value of > 0.98. Notice that the raw spectra data of samples with the same treatment/modification show s.d. of ±1 p.p.m. in chemical shift position. Supplementary Table 8 summarizes the positions fixed for the spectra deconvolution in Lewis acid region (−20 to −58 p.p.m.). For example, during the spectral deconvolution of F-(101) and F-(001), we have fixed two positions of Ti_5c_(101) and Ti_5c_(001) at −31 and −22.5 p.p.m. within ±1 p.p.m. uncertainty. Also, for S-Na-PD, S-Na-(101) and S-Na-(001), the position of Ti_5c_(101) and Ti_5c_(001) were fixed at −25.5 and −34 p.p.m. within ±1 p.p.m. uncertainty.

### Photocatalytic activity test

Comparative catalytic testing was conducted at room temperature, with a constant magnetic stirring to ensure full suspension of the particles. An aqueous MG stock solution (15 mg/L) was used for all photoreactions. A total volume of 20 mg of TiO_2_ sample was dispersed in 100 mL dye stock solution and stirred for 30 min in the dark to allow dye adsorption/desorption to equilibrate. The reaction mixture was then exposed to irradiation with constant stirring in a Luzchem photoreactor fitted with 8 ultraviolet A lamps with non-monochromatic irradiation centred at 350 nm resulting in a combined measured intensity of 4750 lx (Fc = 440).

### Pechmann condensation catalytic activity test

The catalytic activities of TiO_2_ samples were studied for the synthesis of 5,7-dihydroxy-4-methyl coumarin from phloroglucinol and ethyl acetoacetate under solvent-free conditions. In a typical reaction, 5 mmol of phloroglucinol was reacted with 10 mmol of ethyl acetoacetate in the presence of 0.1 g of catalyst. The reaction mixture was kept at 130 °C under reflux for a desired time. On completion of the reaction, the mixture was allowed to cool down to the room temperature followed by the addition of ethanol. The reaction mixture was filtered to separate the catalyst and the filtrate was analysed by liquid chromatography (DIONEX U3000). The yield of 5,7-dihydroxy-4-methyl coumarin was calculated as: Yield (%)=(Obtained weight of product)/(Theoretical weight of product)×100.

### DFT calculations

See Supplementary Note 8 and Supplementary Fig. 23 for the computational details of TMP adsorption energy and corresponding adsorption configuration on various TiO_2_ facets.

### Data availability

The data that support this study are available from the corresponding author on request.

## Electronic supplementary material

Supplementary Information
