# Supplementary material for: Mapping surface-modified titania nanoparticles with implications for activity and facet control
Source: Nat Commun. 2017 Sep 22;8:675. doi: 10.1038/s41467-017-00619-z (PMC5610198; doi:10.1038/s41467-017-00619-z)
Supplement: Supplementary file 1 — Supplementary Information [file 41467_2017_619_MOESM1_ESM.pdf]

Description of Supplementary Files

File Name: Supplementary Information
Description: Supplementary Figures, Supplementary Tables, Supplementary Notes and Supplementary References

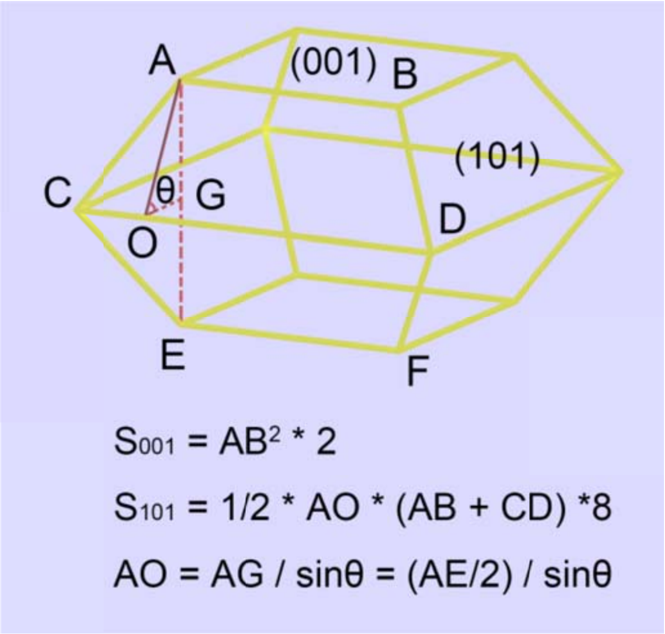

Supplementary Figure 1: Anatase TiO_2 crystal. Simulated shape of the TiO_2 anatase single crystal and the equation for the surface area calculation of (001) and (101) facets (AB and CD are considered of the same value as face length herein; AE is equal to the thickness, θ of 68.3° is the angle between (001) and (101))[1].

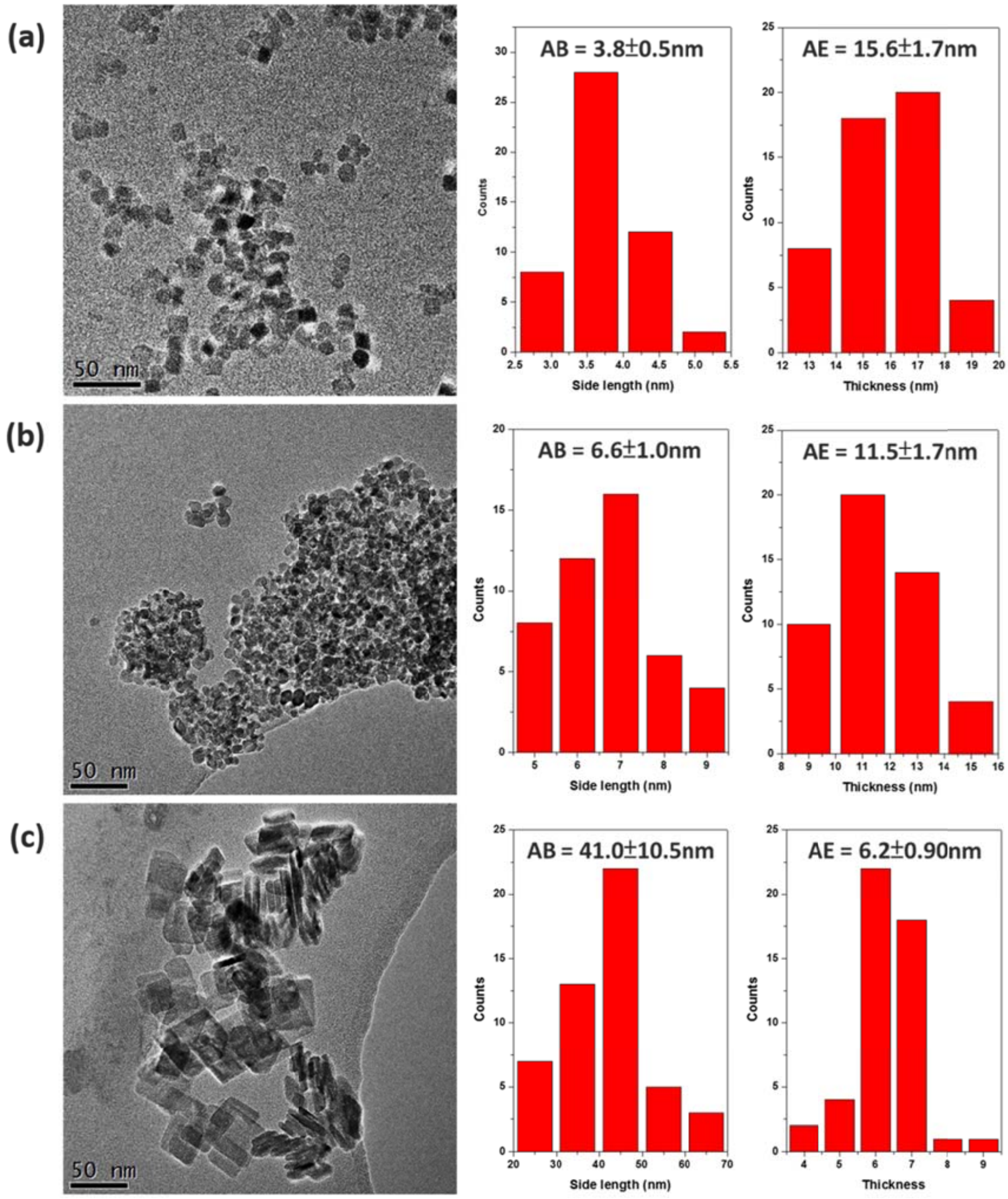

Supplementary Figure 2: TEM images and statistics on particle size. Low magnification TEM images of (a) PD, (b) F-(101), (c) F-(001) and their corresponding AB (face length) and AE (thickness) values (50 particles are used in each histogram).

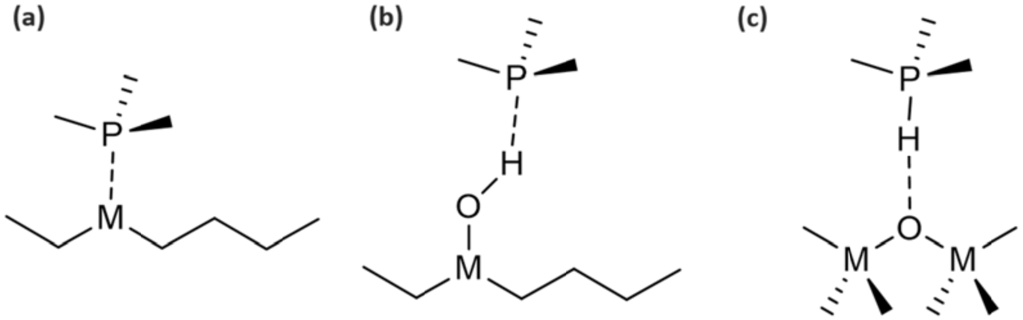

Supplementary Figure 3: Interaction between TMP and metal oxide. TMP molecule interacts (a) with metal cation; (b) with hydroxyl proton (hydrogen bonding interaction); (c) on bridging hydroxyl proton (Brønsted acid, BA) site, the formation of $TMPH^+$ complex).

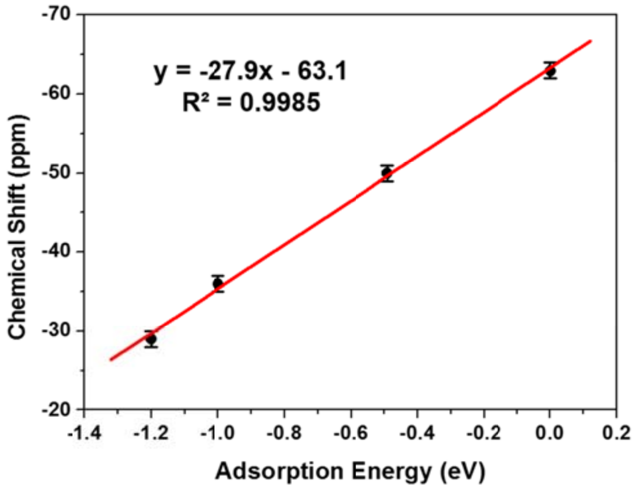

Supplementary Figure 4: Linear correlation between adsorption energy and chemical shift. A linear regression plot by using experimental $\delta^{31}P$ and calculated adsorption energy on various TiO_2 surfaces (also see Supplementary Table 2). The error bar is ± 1 ppm.

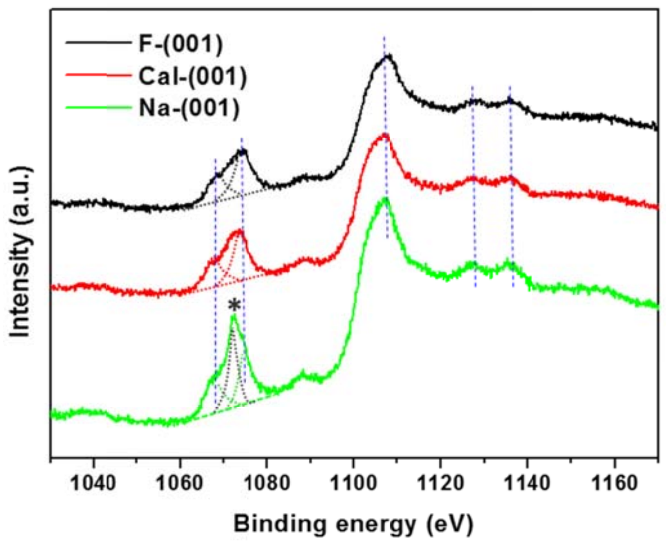

Supplementary Figure 5: XPS Ti Auger measurement. Ti LMM Auger spectra of as-prepared F-(001) TiO_2 samples (marked by dashed blue line) with different treatments (Cal: calcination and Na: NaOH wash). The peak marked with asterisk "*" is Na_{1S} signal.

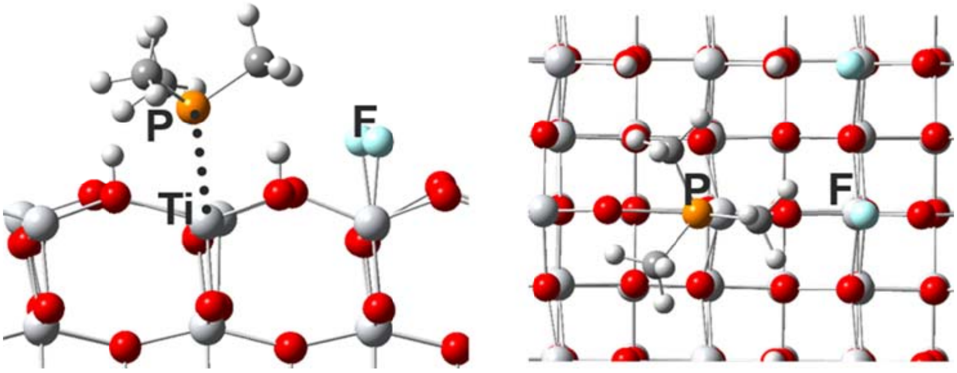

Supplementary Figure 6: DFT calculation. Schematic illustrations of molecular interaction and DFT calculated adsorption energy (E_{ad}) (-1.76 eV) between TMP and Ti_{5C} on fluorine modified (001) facet (F- Ti_{5C}(001)).

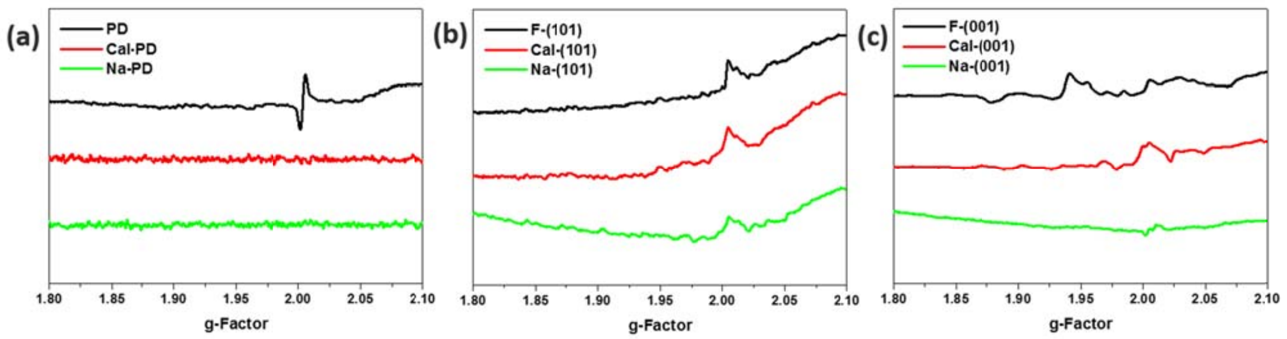

Supplementary Figure 7: EPR measurement. EPR spectra of as-prepared (a) PD, (b) F-(101) and (c) F-(001) TiO_2 samples with different post-treatments (calcination and NaOH wash). In order to make it comparable to NMR results, EPR measurements were carried out at atmospheric pressure and room temperature (the same as NMR measurement).

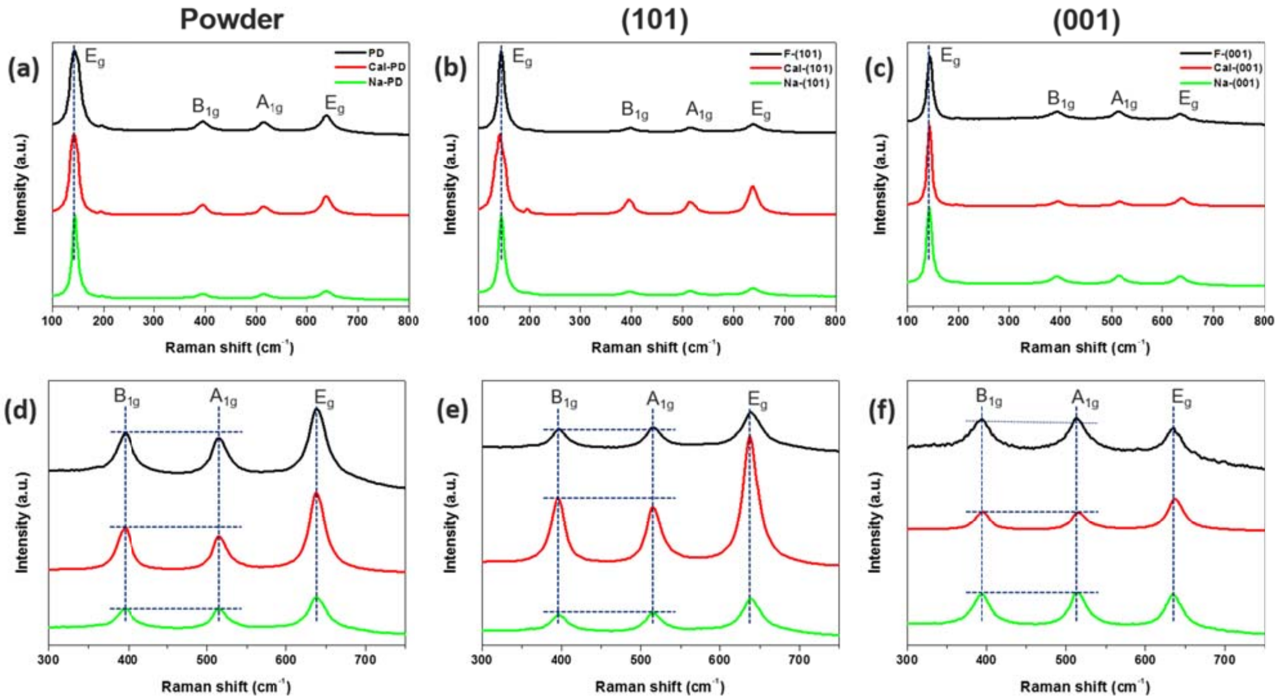

Supplementary Figure 8: Raman measurement. Raman spectra of as-prepared (a) PD, (b) F-(101) and (c) F-(001) TiO_2 samples with different treatment (calcination and NaOH wash). (d), (e) and (f) are enlarged spectra of B_{1g}, A_{1g} and E_g modes.

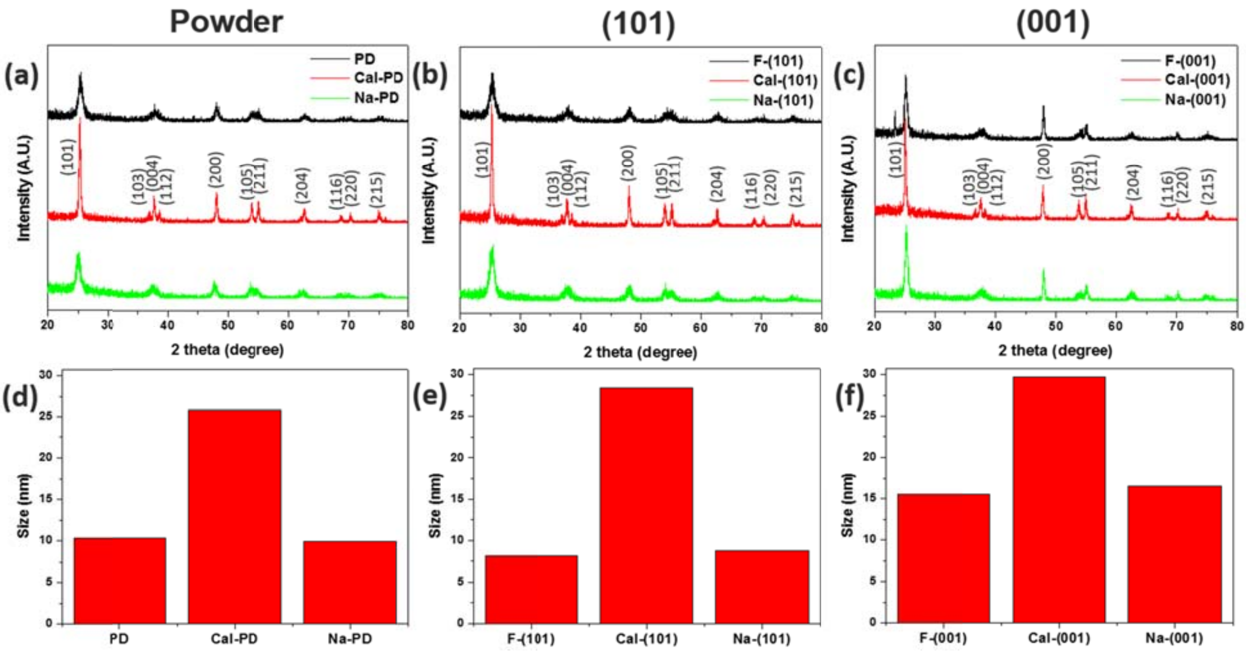

Supplementary Figure 9: XRD and particle size calculation. XRD spectra of as-prepared (a) PD, (b) F-(101) and (c) F-(001) TiO_2 samples with different post-treatments (calcination and NaOH wash). (d), (e) and (f) are corresponding particle size calculated from the full width at half-maximum of the (101) peak using Scherrer equation.

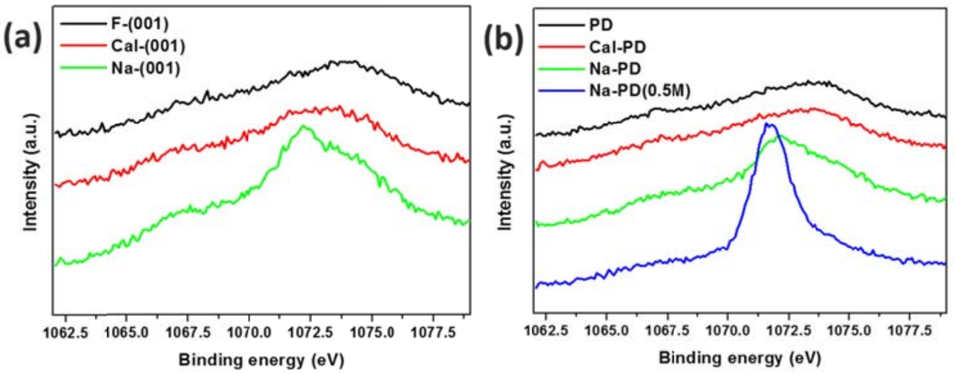

Supplementary Figure 10: XPS measurement in Na_{1S} range. XPS Na_{1S} spectra of as-prepared (a) F-(001) and (b) PD TiO_2 samples with different treatments (calcination and 0.1M NaOH wash).

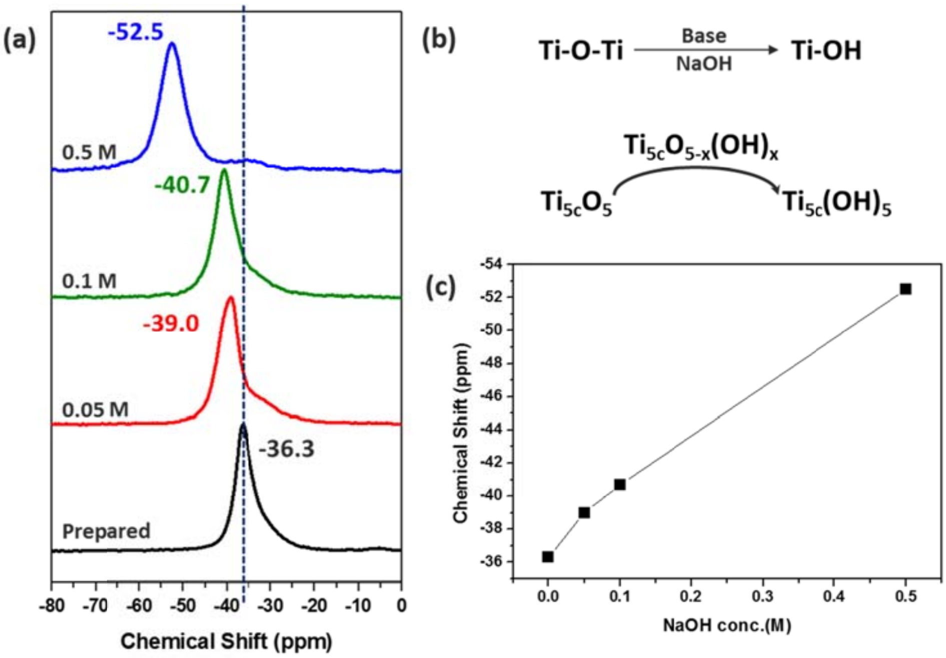

Supplementary Figure 11: NaOH wash induced surface hydrolysis. (a) ^{31}P ssNMR spectra of TMP-adsorbed powder (PD) samples treated with various NaOH concentrations; (b) schematic illustration of the hydrolysis of surface Ti-O-Ti (c) corresponding linear regression plot by using ^{31}P chemical shift and NaOH concentration.

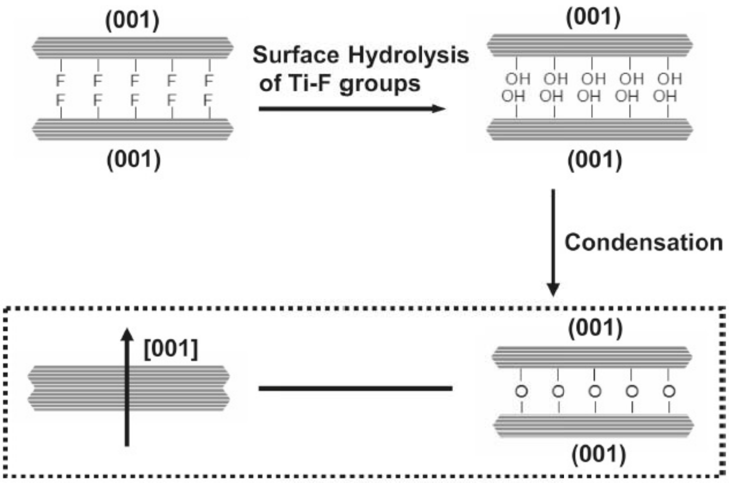

Supplementary Figure 12: Calcination-induced aggregation. The growth mechanism of stacked anatase TiO_2 nanosheets dominated by (001) facets at their interfacial regions proposed by Yang *et al.*[2].

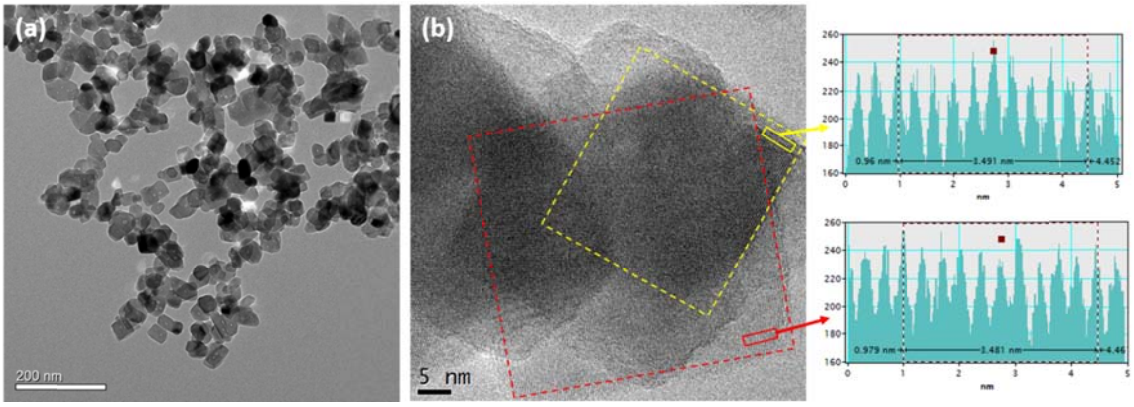

Supplementary Figure 13: TEM images of Cal-(001) sample. (a) low and (b) high magnification TEM images of Cal-(001). Ten lattice fringes of two fused Cal-(001) (red and yellow square) were measured to be 3.491 nm (yellow) and 3.481 nm (red) in length. The lattice spacing was then calculated to be 0.3491 and 0.3481 nm, both corresponds to the (101) plane of anatase TiO_2 structure and indicates the longitudinal stacking of TiO_2 nanosheets.

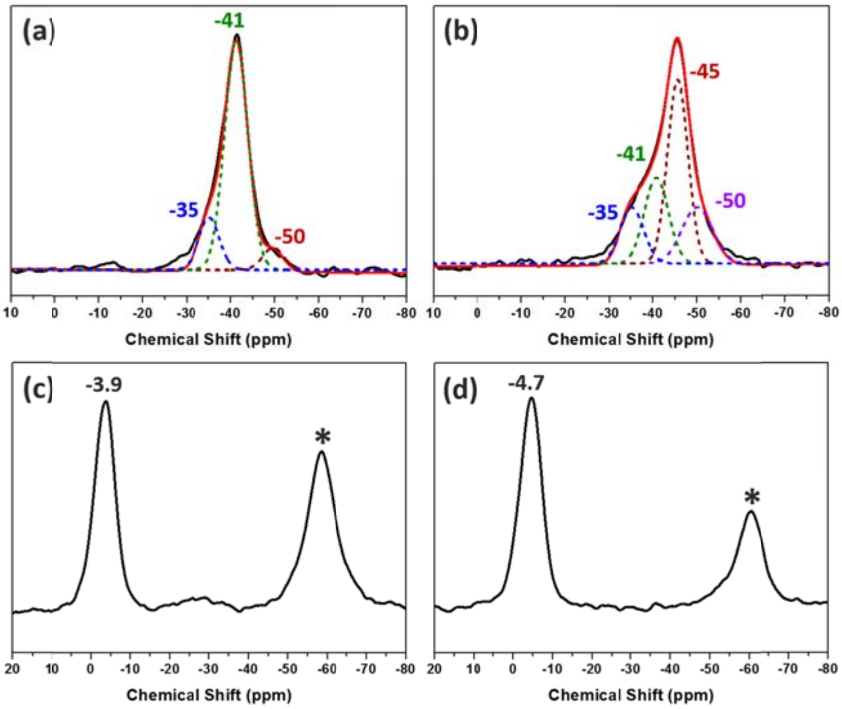

Supplementary Figure 14: ^{31}P ssNMR spectra of calcined TiO_2 samples. ^{31}P ssNMR spectral deconvolution of TMP-adsorbed TiO_2 samples treated with calcination (a) Cal-PD, (b) Cal-(001) and their following sulfation treatment (c) S-Cal-PD, (d) S-Cal-(001). *: physisorbed TMP (~-61 ppm).

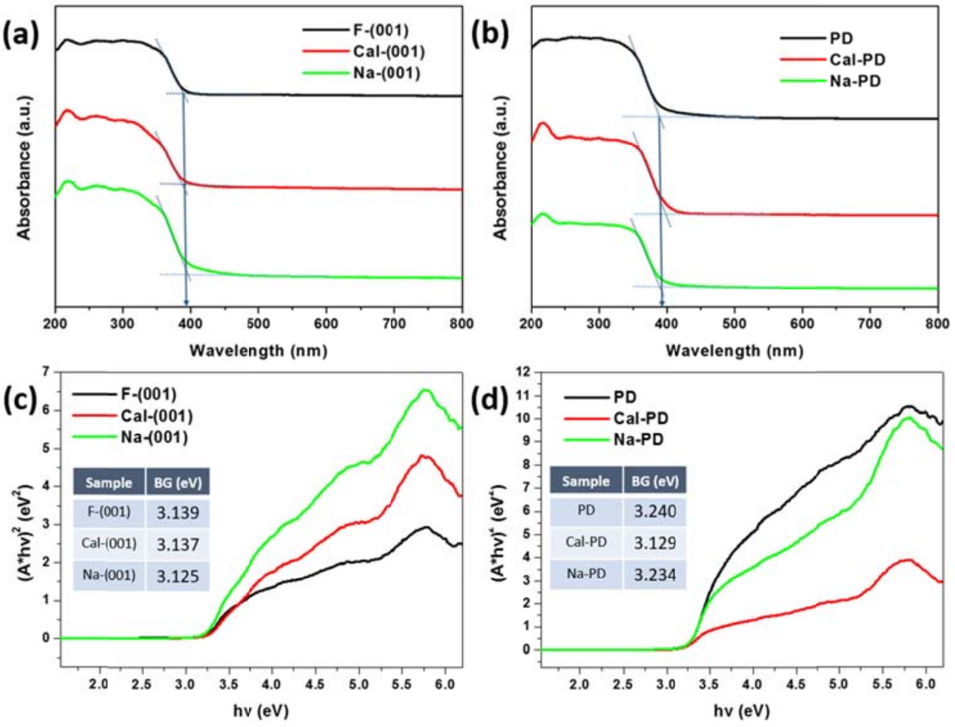

Supplementary Figure 15: UV-vis measurement. UV-visible absorption spectra of samples with preferential exposed (001) facet (a) F-(001), Cal-(001), Na-(001) and samples with preferential exposed (101) facet (b) PD, Cal-PD, Na-PD. (c) and (d) are the corresponding Tauc plots (BG: bandgap). Only a small difference in their bandgap values was observed in the samples after calcination or NaOH wash and no direct correlation of the width of bandgap with the photocatalytic activity can be found.

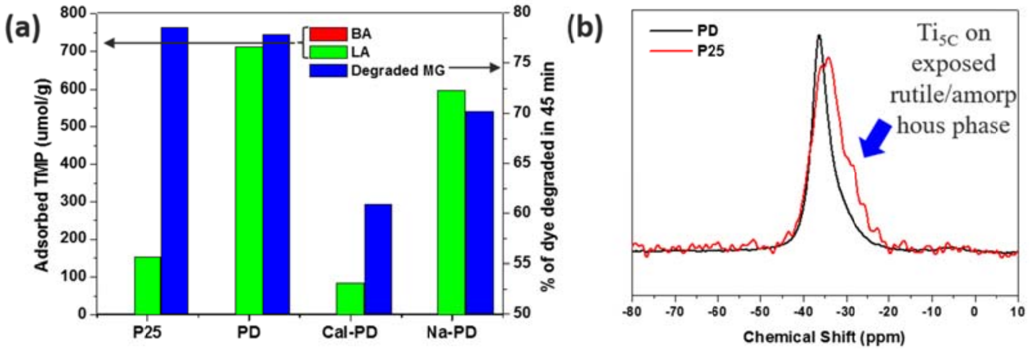

Supplementary Figure 16: Photoactivity for samples preferential exposed (101) facet. (a) Comparison of BA and LA for photocatalytic decomposition rate of MG dye in 45 min over PD samples with various treatments. Polycrystalline Degussa P25 possessing both anatase and rutile phases is also compared here. (b) ^{31}P MAS NMR spectra of TMP-adsorbed PD and P25.

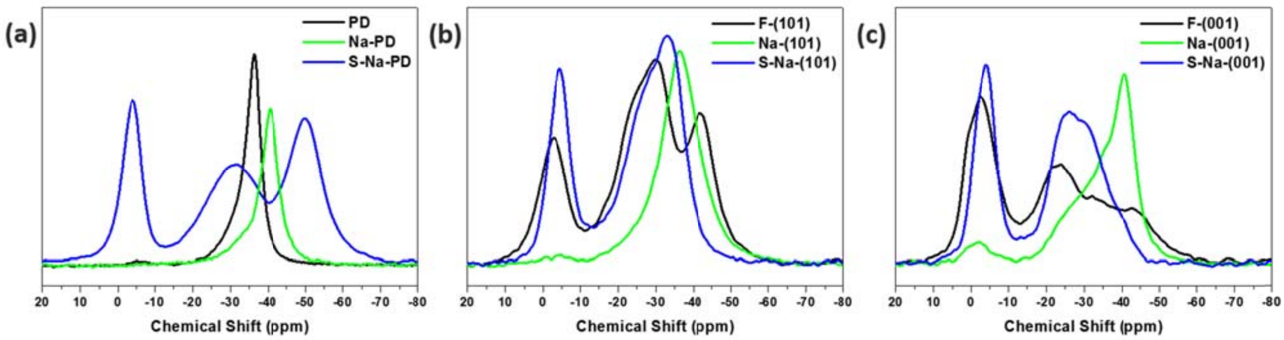

Supplementary Figure 17: Adsorbate-dependent NMR spectra (NaOH wash). The electronic effect imposed by different adsorbates during sequential treatments/modifications to surface cation. on as-prepared (a) PD, (b) F-(101) and (c) F-(001) (black line) with 0.1M NaOH treatment (green line) followed by sulfate modification (blue line).

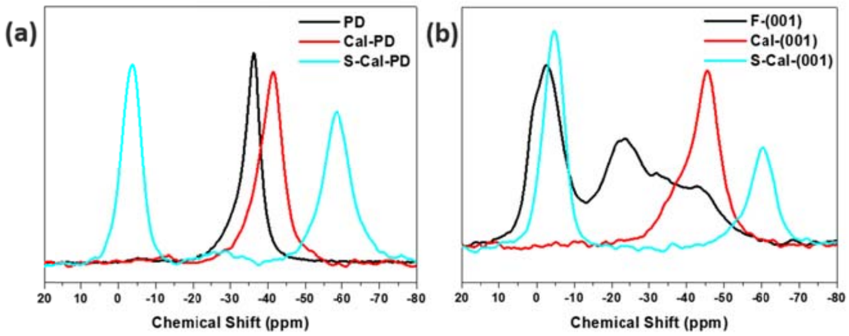

Supplementary Figure 18: Adsorbate-dependent NMR spectra (calcination). The electronic effect (chemical shift) imposed by different adsorbates during sequential treatments/modifications to surface cation on as-prepared (a) PD, (b) F-(001) (black line) with calcination treatment (red line) followed by sulfate modification (cyan line).

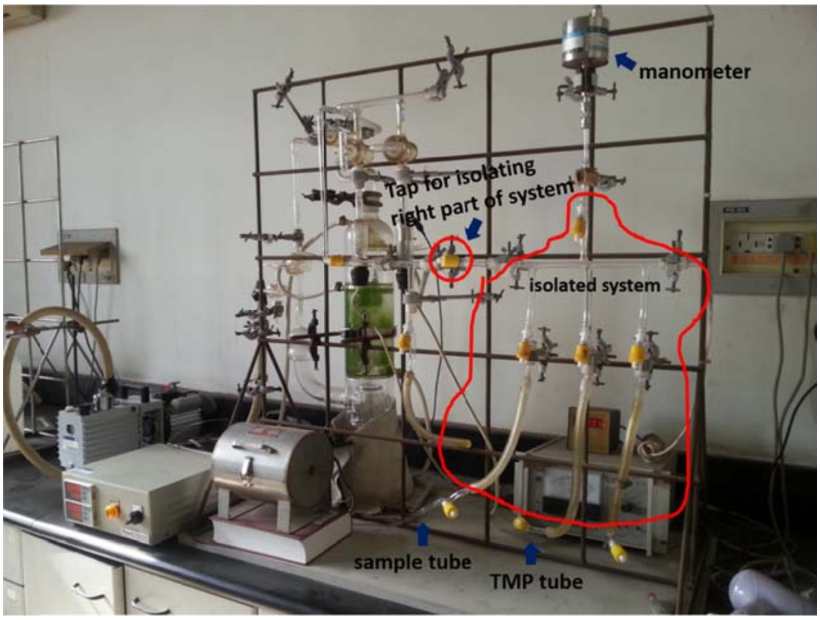

Supplementary Figure 19: The system setup for TMP-adsorption experiment.

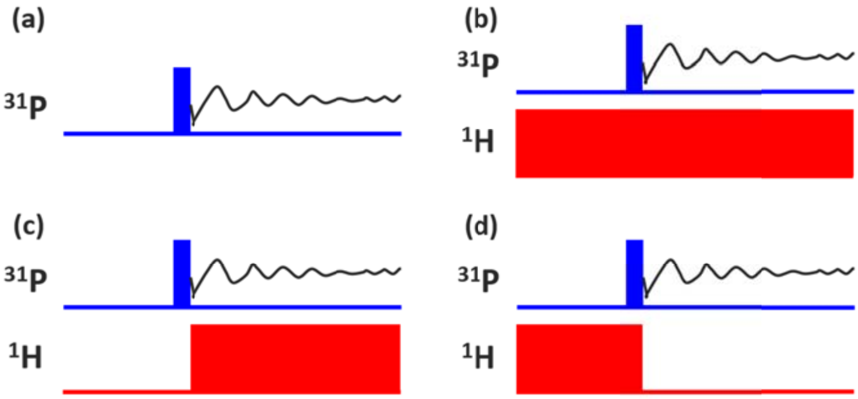

Supplementary Figure 20: Modes of broadband heteronuclear decoupling. (a) One-pulse sequence (no decoupling), (b) 100% duty cycle proton decoupling (for proton decoupling and nuclear overhauser effect (NOE) enhancement), (c) inverse gated proton decoupling (no NOE but proton decoupling) and (d) gated proton decoupling (proton coupling and NOE enhancement).

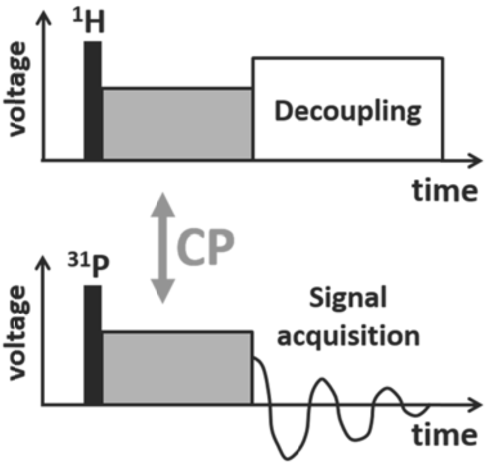

Supplementary Figure 21. Pulse sequence of $^{1}H \rightarrow ^{31}P$ cross polarization MAS solid-state NMR.

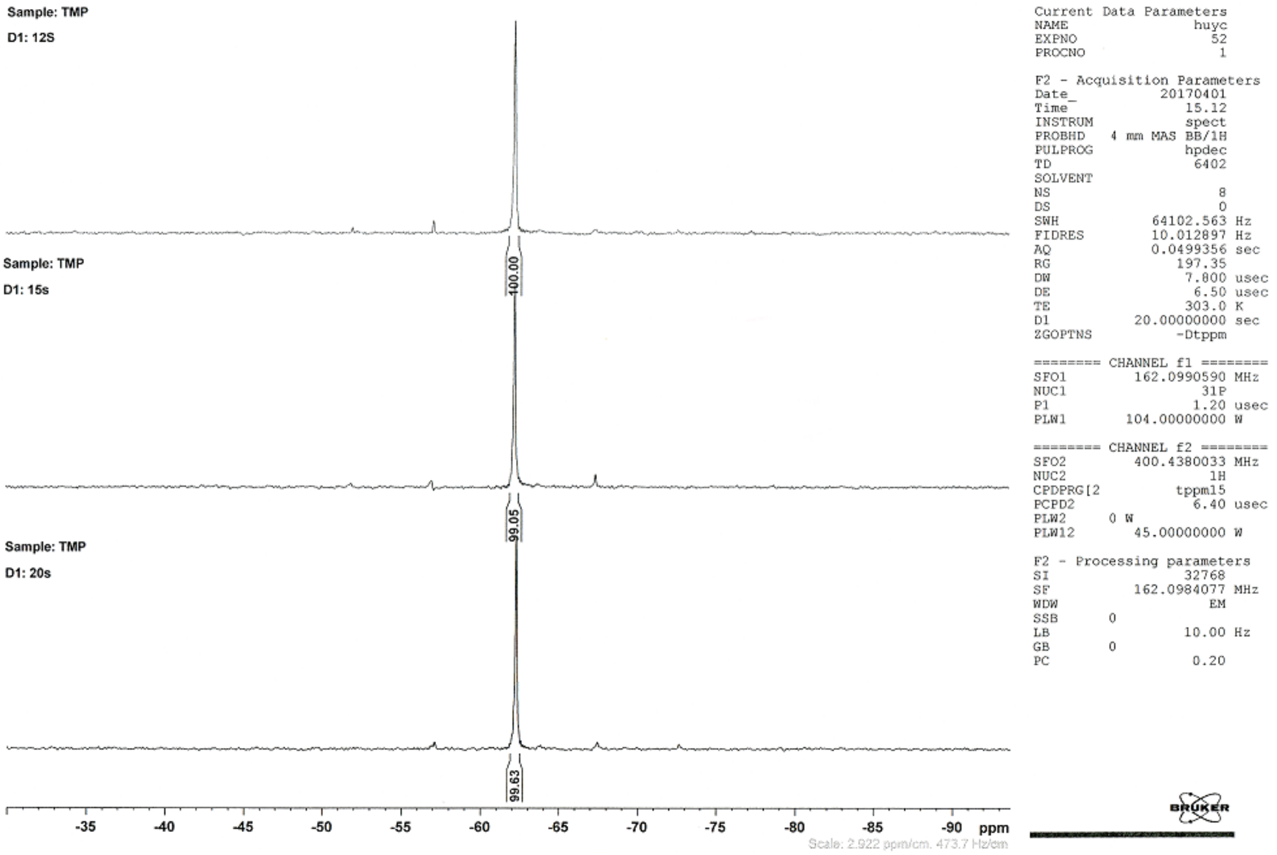

Supplementary Figure 22: NMR sequences with different delay time. ^{31}P MAS NMR spectra of pure TMP obtained at various delay time (12, 15 and 20 s).

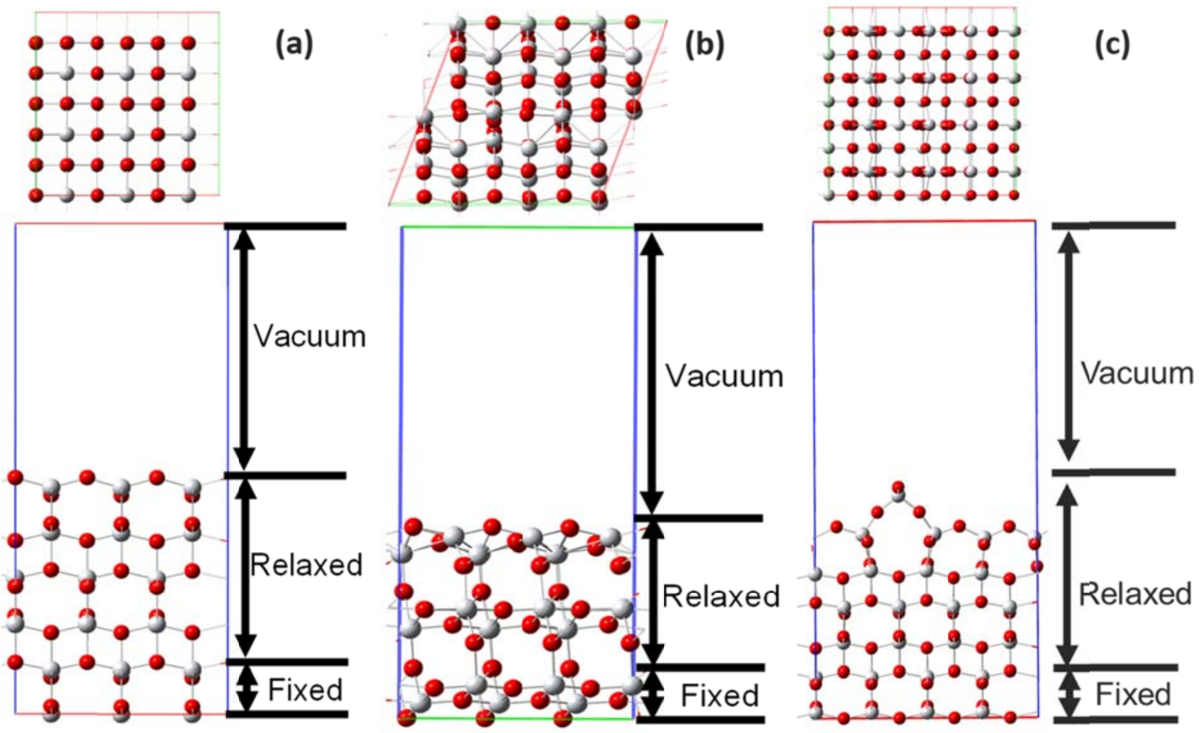

Supplementary Figure 23: DFT simulation. The calculation models of (a) a-TiO_2(001), (b) a-TiO_2(101) and (c) a-TiO_2Re-(001) from top-view (upper row) and side-view.

Supplementary Table 1: Sample preparation conditions and the percentage of exposed (101)/(001) facets.

Sample	Solvent	AB (Face length, nm)	AE (Thickness, nm)	% (101)	% (001)
Powder	6 mL H_2O	3.8 ± 0.5	15.6 ± 1.7	89.8	10.2
F-(101)	2 mL HF/4 mL H_2O	6.6 ± 1.0	11.5 ± 1.7	78.9	21.1
F-(001)	6 mL HF	41.0 ± 10.5	6.2 ± 0.9	24.6	75.4

Supplementary Table 2: Experimental $\delta^{31}P$ and calculated adsorption energy of TMP on various TiO_2 surfaces.

Structure	$E_{adsorption}$ (eV)_cal.	$\delta^{31}P$ (ppm)_expt.
TMP molecule	0.00	-63 ± 1
Ti_{5C}RC-(001)	-0.49	-50 ± 1
Ti_{5C}(101)	-1.00	-36 ± 1
Ti_{5C}(001)	-1.20	-29 ± 1

Supplementary Table 3: Atomic ratios of TiO_2 samples evaluated by XPS with different post-treatments (calcination and NaOH wash).

Powder	Ti : O : F ratio	(101)	Ti : O : F ratio	(001)	Ti : O : F ratio
PD	1 : 1.987 : 0.000	F-(101)	1 : 1.915 : 0.180	F-(001)	1 : 1.820 : 0.400
Cal-PD	1 : 1.961 : 0.000	Cal-(101)	1 : 1.980 : 0.000	Cal-(001)	1 : 1.976 : 0.000
Na-PD	1 : 2.050 : 0.000	Na-(101)	1 : 1.955 : 0.000	Na-(001)	1 : 1.976 : 0.124

Supplementary Table 4: EPR quantitative information of TiO_2 samples with different treatment (calcination and NaOH wash).

Powder	g~2.0 (counts/g)	(101)	g~2.0 (counts/g)	(001)	g~2.0 (counts/g)	g~1.95 (counts/g)
PD	7.811×10^{14}	F-(101)	1.276×10^{15}	F-(001)	2.576×10^{15}	3.442×10^{15}
Cal-PD	-	Cal-(101)	1.181×10^{15}	Cal-(001)	1.368×10^{15}	-
Na-PD	-	Na-(101)	9.408×10^{14}	Na-(001)	9.020×10^{14}	-

Supplementary Table 5: XPS Atomic ratios and EPR g value at 2.0 of TiO_2 samples extracted from Supplementary Table 3 and 4.

Sample	XPS Ti : O : F ratio	EPR g~2.0 (counts/g)
F-(001)	1 : 1.820 : 0.400	2.576×10^{15}
F-(101)	1 : 1.915 : 0.180	1.276×10^{15}
Na-(001)	1 : 1.976 : 0.124	9.020×10^{14}

Supplementary Table 6: BET surface area data of TiO_2 samples.

Powder	BET (m^2/g)	(101)	BET (m^2/g)	(001)	BET (m^2/g)
PD	123.3	F-(101)	163.0	F-(001)	83.0
Cal-PD	15.8	Cal-(101)	40.8	Cal-(001)	29.9
Na-PD	145.4	Na-(101)	152.4	Na-(001)	85.1
S-Na-PD	107.0	S-Na-(101)	77.3	S-Na-(001)	67.4

Supplementary Table 7: Mixtures of $NH_4H_2PO_4$ and $NaNO_3$ with different ^{31}P concentration and their corresponding quantitative ^{31}P NMR results.

Mixture	The weight percent of $NH_4H_2PO_4$	The total weight of measured sample (mg)	The relative number of ^{31}P	The relative area of ^{31}P signal
A	100.0	115.1	100	100
B	74.6	115.4	75	77
C	48.8	122.7	52	53

Supplementary Table 8: Summary of the positions fixed for the spectra deconvolution in LA region (-20 ppm to -58 ppm).

**Positions fixed in for deconvolution**		
**Peak 1**	**Peak 2**	**Peak 3**
F-(001) & F-(101)		
-22.5	-31	-42.5
Na-(001) & Na-(101)		
-28	-36.5	-41
Cal-(001) & Cal-PD		
-35	-41	-50
S-Na-(001) & S-Na-(101) & S-Na-PD		
-25.5	-34	-

Supplementary Note 1: ^{31}P MAS NMR analysis of TMP adsorbed on metal oxide.

Pioneered by Lunsford and co-workers, TMP was first adopted as a probe molecule to characterize the acidity of zeolite based on the observed ^{31}P chemical shift ($\delta^{31}P$)[3]. Thereafter, the technique has been widely utilized for acidity characterization of various solid acid catalysts[4]. Supplementary Fig. 3 shows three scenarios of interactions between TMP and metal oxide: (a) with metal cation LA center; (b) with hydroxyl proton LA center (hydrogen bonding interaction); (c) on bridging hydroxyl proton (Brønsted acid, BA) site, the formation of $TMPH^+$ complex). The $\delta^{31}P$ of adsorbed TMP spans over a wide range (-20~-58 ppm) when interacting with various metal cations on different solid acids (i.e. case (a)), whereas a $TMPH^+$ ionic complex formed when a TMP molecule adsorbs onto a bridging hydroxyl proton tends to give rise to a ^{31}P resonance in a much narrower range of -2 to -5 ppm (i.e. case (c)). Therefore, Brønsted (proton donor) and Lewis acid (electron acceptor) sites presented in a solid acid catalyst can be readily distinguished using ^{31}P ssNMR of adsorbed TMP. On the other hand, TMP on an isolated hydroxyl proton surface usually gives a signal at higher field (~-61 ppm, i.e. case (b)).

Supplementary Note 2: EPR study of as-prepared PD, F-(101) and F-(001) samples with different treatments.

As shown by Wöll's group[5] that the decrease of the saturation coverage of protons of metal oxide was attributed to the generation of V_o by recombination of H atoms with OH species (i.e. thermal desorption of water). To ensure all measurements (i.e. EPR/Raman/ssNMR) were carried out under consistent environment, we herein carried out EPR measurement at ambient temperature rather than 80K[6] or 130K[7] as previously reported. The signal at g value around 2.0 has commonly been assigned to the unpaired electrons deeply trapped in V_o via adsorbed oxygen species from air (O_2^-) and the signal at g=1.95 represents unpaired electrons trapped by surface/subsurface paramagnetic Ti^{3+} center. Corresponding EPR quantitative information of g value at 2.0 and 1.95 of samples with different post-treatments (calcination and NaOH wash) is summarized in Supplementary Fig. 7 and Supplementary Table 4. Only F-(001) with highest surface F concentration reveals a dominant signal at g = 1.95, while this peak disappeared after either calcination or NaOH wash. On the other hand, the quantitative result of V_o from g ~ 2.0 also decreases with the removal of surface fluorine. Both results indicate the formation of oxygen vacancy is positively related to the concentration of surface fluorine.

Due to the long electron escaping depth of XPS (up to 10 nm), it is not a truly surface analysis (detection limit ~ 0.1% atom). As a result, oxygen vacancies beyond the topmost layer of TiO_2 can be included. In a first glance of the data, it may not be easy to see the direct and consistent correlation on the deviation of O/Ti (oxygen vacancies) with the introduction of surface F from (F/Ti ratio). However, according to the XPS data summarized in Supplementary Table 3, only three TiO_2 samples (i.e. F-(001), F-(101) and Na-(001)) showed the presence of fluorine (it must be on the top upper layer). Supplementary Table 4 shows the corresponding quantitative EPR measurements at g ~ 2.0 (unpaired electrons deeply trapped in surface oxygen vacancy, V_o via adsorbed oxygen from air as O_2^-) over the same samples. To demonstrate the clear correlation between surface F with surface V_o over these samples, Supplementary Table 5 is created. As seen from the ratios of O/Ti of the F-(001), F-(101) and Na-(001) samples, which give the increasing values from 1.820, 1.915 to 1.976. They match with the simultaneous decrease in F/Ti ratios from 0.40, 0.18 to 0.124, respectively. The result suggests that the decreasing order of oxygen vacancies (deviated from the theoretical ratio of O/Ti = 2 in pure surface TiO_2) corresponds to the decrease in the surface fluorine contents (electron withdrawing property of F). Thus, the F-(001) with the highest F/Ti ratio possesses the highest V_o concentration (O/Ti = 1.820), while the Na-(001) with the lowest F/Ti ratio possesses the lowest V_o concentration (O/Ti = 1.976).

Supplementary Note 3: Raman study of as-prepared F-(001), F-(101) and PD samples with different treatments.

According to previous literature[8-10], the removal of surface fluorine can be monitored by Raman spectroscopy. It has been shown that the surface attached fluorine changes both "symmetry of Ti-O-Ti" and "coordination of surface Ti atom", resulting in the "shift of low-frequency E_g" and "weakening of B_{1g} (cf. A_{1g})" after fluorine removal. However, from our experiment result, only a marginal shift of low-frequency E_{1g} is observed (Supplementary Fig. 8a-c). Calcination treatment (Supplementary Fig. 8d-f), as expected, results in $B_{1g} > A_{1g}$, while the intensity $B_{1g} = A_{1g}$ case is observed herein on NaOH washed samples. These observations give hints the performances on the removal of surface F (calcination or NaOH wash) which showed the change in the coordination of surface Ti atom.

Supplementary Note 4: Na^+ ions left on TiO_2 surface after NaOH wash.

Detailed XPS scanning in the Na_{1S} region has been carried out over the samples with preferential exposure of (001) facet (i.e. (F-(001)) and (101) facet (i.e. PD, prepared without HF). No Na_{1S} signal at 1072 eV for both F-(001) and PD and their corresponding calcination samples (i.e. Cal-(001) and Cal-PD) is detected (Supplementary Fig. 10). Notice that the broad signals at 1067 eV and 1073 eV are the Ti LMM Auger signals. A very small trace of Na_{1S} signal can be marginally detected for both Na-(001) and Na-PD (green line) after the samples were pre-treated with 0.1M NaOH, followed by rinsing with DI water several times (> three times). This suggests majority of Na^+ ions had been removed without interfering to the measured chemical shift values of TMP by NMR. The Na^+ on surface can only be quantifiable by XPS for the sample treated with 0.5M NaOH (~6.29%, blue line)[11].

Supplementary Note 5: Photocatalytic activity of TiO_2 with preferential exposed (101) facet.

In addition to (001) facet, similar result was also obtained for samples with preferential exposed (101) facet (i.e. PD, Cal-PD and Na-PD). As shown in Supplementary Fig. 16a, the photocatalytic activity is correlated to the overall concentration of Lewis acid (LA) sites: 712.1 µmol/g of PD > 596.9 µmol/g of Na-PD > 84.8 µmol/g of Cal-PD. We also carried out the photocatalytic testing on Degussa P25 for comparison. P25 with less than one fourth LA concentration (151.7 umol/g) to that of PD (712.1 umol/g) exhibit comparable photocatalytic activity. The large difference in LA concentration could be attributed to their surface area: PD (123.3 m^2/g) > P25 (40.3 m^2/g). However, the similar photocatalytic activity implies there is another factor overrides the total LA concentration in P25 case. It is noted that all TiO_2 samples compared in this study are single crystalline 100% anatase structure with different ratio of (001) and (101) surface. While P25 is a well-known polycrystalline TiO_2 nanoparticle containing more than 70% anatase with a minor amount of rutile and sometimes a small amount of amorphous phase. The ratio of crystalline composition (anatase to rutile) of P25 has been found changed from time to time even though they are from the same package[12]. Similar fluctuations of crystalline composition of P25 has also been reported before[13,14]. The intrinsic interfaces between those anatase and rutile domains have been demonstrated greatly improve charge separation efficiency because of the well-formed type-II band alignment at the anatase and rutile interface[15]. ^{31}P MAS NMR study of TMP-adsorbed Degussa P25 (Supplementary Fig. 16b) shows a main signal of surface anatase $Ti_{5c}(101)$ at -35 ppm as our PD sample, while the shoulder with irregular shape appearing at lower field can be attributed to Ti_{5c} from surface amorphous or rutile phase. Considering the factors from both inside (charge separation,

poly/single crystallinity) and outside (Ti_{5c} from anatase/rutile/amorphous) particle, it is thus difficult to study the correlation between surface and catalytic result by a simple comparison of catalytic result between polycrystalline P25 and all other anatase samples in this study. However, this is a good example to illustrate the importance of factor isolation from particle side (both intrinsic and extrinsic) in an aim to correlate the corresponding catalytic activity. By carefully tuning those factors one at a time, we believe those different interpretations and frequently disagreements amongst researchers can be largely avoided.

Supplementary Note 6: The system setup for preparation of TMP-adsorbed samples.

About 150 mg of TiO_2 was placed in a home-made glass tube and activated at 150 ^oC for 2 h under vacuum (10^{-1} Pa) to ensure maximum adsorption of TMP molecules. After cooling down to room temperature, the system connecting TMP tube and sample tube (Supplementary Fig. 19) was isolated from the left part of vacuum system before the introduction of TMP molecules. 300 μmol/catalyst g (calculated by the pressure and volume of isolated system) of TMP was then introduced into this system. Wait for ~10 min until the pressure of this isolated system reach a plateau, which means the equilibrium between TMP and catalyst surface has been achieved. The tap to TMP and sample tubes were then closed before the removal of extra TMP molecules by left vacuum system. These steps were repeated three times to ensure the fully adsorption of TMP on catalyst surface. The sample tube was then flame sealed for storage and transferred to Bruker 4 mm ZrO_2 rotor with a Kel-F endcap in a glove box under nitrogen atmosphere before NMR measurement.

Supplementary Note 7: ^{31}P MAS NMR experiments.

Solid state magic angle spinning (MAS) NMR experiments were carried out using a Bruker Avance III 400WB spectrometer at room temperature. To remove the effect of proton spins on ^{31}P spectra, a strong radio frequency field (B) is usually applied in a pulsed at the resonance frequency of the non-observed abundant spins (^{1}H herein) which contribute to the coupling of both spin species. If B is strong such that spins of ^{1}H is flipped rapidly compared with the spin-spin interactions, the interaction is averaged to zero and consequently the excess broadening is zero. The high power decoupling (HPDEC) was thus used for the quantitative ^{31}P analysis. Considering the long relaxation time of ^{31}P nuclei in NMR experiment, we used 30° pulse with the width of 1.20 μs, 15 s delay time. The radiofrequency for

decoupling was 59 kHz. The spectral width was 400 ppm, from 200 to −200 ppm. The number of scanning was 800. The ^{31}P chemical shifts were reported relative to 85% aqueous solution of H_3PO_4, with $NH_4H_2PO_4$ as a secondary standard (0.81 ppm). The quantitative analysis of adsorbed TMP molecules was calculated according to the calibration line established by running standard samples with various adsorbed TMP concentration.

A simple one-pulse sequence as shown in Supplementary Fig. 20a may generally be used to quantify ^{31}P from the signal intensity in solid-state NMR. However, this application strongly subjects to the environment of ^{31}P nucleus used. Regarding to the probe molecule, the trimethylphosphine (TMP), three ^{1}H are close to ^{31}P in space causing a strong heteronuclear dipole-dipole coupling interaction. This dipole-dipole coupling interaction is much stronger than the J-coupling interaction normally observed in liquid NMR. Thus, the former leads to severe broadening of the ^{31}P peaks in TMP study. Therefore, it results serious overlapping of neighboring peaks and increases the difficulties in the peak assignments. This dipole-dipole interaction can be efficiently removed by introducing the second frequency for ^{1}H decoupling. However, if the ^{1}H decoupling is applied during the entire duration of the experiment (recycle delay and data acquisition) (Supplementary Fig. 20b), the nuclear overhauser effect (NOE) will enhance the signals from certain phosphorus disproportionately, leading to non-quantitative spectra. To remove the interference of NOE from quantitative analysis, we adopted the inverse gated decoupling (Supplementary Fig. 20c): the decoupling is on only during the acquisition period, to suppress NOE and obtain a quantitative result. Compared with one-pulse ^{31}P MAS NMR experiment, a continuous irradiation is applied to the ^{1}H channel during the acquisition time in our HPDEC (high power decoupling) MAS NMR experiment.

As this HPDEC sequence can efficiently eliminate the influence of dipole-diploe coupling interactions from ^{1}H and NOE effect, it has actually been widely employed in MAS NMR measurements, e.g. for the quantitative evaluations of Brönsted/Lewis acid sites on TMP adsorbed microporous zeolites reported in literature (H-mordenite[16], H-ZSM-5[17]), mesoporous molecular sieves (SBA-15 and MCM-41[18]) and metal oxide nanoparticles (TiO_2[19], Niobates[20], ZnO[21]). To further demonstrate the HPDEC sequence in our study can be used quantitatively, $NH_4H_2PO_4$ and $NaNO_3$ were physically mixed with three different weight percents (i.e. 100%, 74.6% and 48.8% for $NH_4H_2PO_4$, Supplementary Table 7). Corresponding ^{31}P HPDEC MAS NMR results are also summarized in the Table. By normalizing the number of the ^{31}P nuclei in pure $NH_4H_2PO_4$ (i.e. mixture A) and its NMR

peak intensity as 100, the relative ^{31}P peak areas of their mixture B and C were found to match very well with the numbers of ^{31}P nuclei in each mixture.

Although cross polarization (CP) technique has been widely employed in solid-state NMR to enhance the signal of nuclei with low gyromagnetic ratio or long T_1 relaxation. For the case of TMP, the abundant nucleus is ^{1}H and the observed nucleus is ^{31}P. If the abundant ^{1}H is excited, and its energy is transferred to the observed ^{31}P by using a CP on both channels (Supplementary Fig. 21). The ^{31}P signal intensity can thus be enhanced by exploiting the polarization of the nearby proton nuclei. Since this process involves transfer from ^{1}H to ^{31}P in the solid state, the number and distance of proton nearby could significantly vary ^{31}P signal intensity. However, as the surface probe molecule for solid metal oxide, the number and distance of proton around ^{31}P (TMP) vary with its interactions with different surface features. As shown in Supplementary Fig. 3, the TMP molecule can bind to metal cation (a), isolated (b) and bridging (c) hydroxyl proton. Both the bottom cases (especially for the bottom right case with chemical bond formation between ^{1}H and ^{31}P) can give stronger ^{31}P intensity as an additional proton in a close proximity (cf. upper case). As CP could lead to variation in signal intensities with multiple ^{31}P environments, we thus adopted HPDEC rather than CP in this paper for the quantification of various surface features.

As we know, the T_1 for adsorbed TMP should be shorter than pure TMP as a result of the additional interactions between adsorbed TMP and solid adsorbent. Under the same acquisition parameters, if a delay time is sufficiently long enough for pure TMP sample, it will be enough for bound TMP on adsorbents and can be employed for the ^{31}P MAS NMR experiments in this paper. To shorten the delay time and obtain a better signal-to-noise ratio in a given time, we have used 30° pulse with a pulse width of 1.2 μs in the ^{31}P MAS NMR experiments for both the pure TMP and also the adsorbed TMP in this paper. First, we introduced a fixed quantity of TMP into a home-made glass tube, which fitted into a 4 mm Bruker zirconia rotor, with the help of liquid nitrogen in a vacuum line. Then, we chose 12, 15 and 20 s as the delay times while keeping other parameters unchanged. ^{31}P MAS NMR spectra were recorded accordingly and can be seen in Supplementary Fig. 22. The parameters shown in the right side of the picture were the acquisition and processing parameters for those spectra when the delay time of 20 s was chosen as an example. We defined the peak area in ^{31}P MAS NMR spectrum obtained at a delay time of 12 s as 100, the peak area in the other two spectra obtained at a delay time of 15 s and 20 s, was found to be 99 and 100, respectively. So, a delay time of 15 s was sufficiently long enough for pure

TMP in the present acquisition conditions, and was therefore chosen for the ^{31}P MAS NMR experiments in this paper.

Supplementary Note 8: Computational details.

In DFT calculations, we employed projector-augmented waves (PAW)[22,23] generalized gradient approximation (GGA)[24]. In the plane wave calculations, cutoff energy of 500 eV was applied and was automatically set by the total energy convergence calculation for anatase $TiO_2(001)$ [a-$TiO_2(001)$], anatase $TiO_2(101)$ [a-$TiO_2(101)$] and anatase TiO_2 with (1x4) reconstructed (001) [a-TiO_2Re-(001)] slab systems. DFT simulations were then performed based on a-$TiO_2(001)$, a-$TiO_2(101)$ and a-TiO_2Re-(001) slab systems shown in Supplementary Fig. 23. Initially, the primitive unit cell of TiO_2 was constructed to consist of tetragonal anatase TiO_2 structure containing eight O atoms with four Ti atoms; the system was then allowed to reach its lowest energy configuration by a relaxation procedure. The k-point grid determined by the Monkhorst-Pack method was 7 × 7 × 3 for bulk calculations in this study. The calculated lattice parameters of TiO_2 were 3.776 × 3.776 × 9.486 Å, which was in good agreement with the experimental value (3.785 × 3.785 × 9.514 Å)[25].

For the modeling of a-$TiO_2(001)$, we adopted a slab containing six Ti-O units. The surface was constructed as a slab within the three dimensional periodic boundary conditions. This model was separated from their images in the z direction perpendicular to the surface by a 14 Å vacuum layer (the x and y directions being parallel to the surface). The bottom three layers were kept fixed to the bulk coordinates; full atomic relaxations were allowed for the top six layers. For these calculations, a 3 × 3 × 1 *k*-Point mesh was used in the 4 × 4 super cell. A suitable dimension of supercell (11.328 × 11.328 × 26.255 $Å^3$) was found to perform the adsorption of trimethylphosphine (TMP) on a-$TiO_2(001)$. The atoms in the cell were allowed to relax until the forces on unconstrained atoms were less than 0.02 eV/Å. The adsorption energy in TMP-a-$TiO_2(001)$ system, E_{ad}, is defined as the sum of interactions between the capping molecule and slab system, and it is given as $E_{ad} = E_{total} - E_{a-TiO_2(001)} - (E_{TMP})$, where E_{total}, $E_{a-TiO_2(001)}$ and E_{TMP} are the energy of total system, a-$TiO_2(001)$ slab and TMP molecule, respectively. Notice that the negative sign of E_{ad} corresponds to the energy gain of the system due to molecular adsorption. The calculation of TMP-a-$TiO_2(101)$ and TMP-a-TiO_2Re-(101) system were carried out similarly.

Supplementary References:

[1] Hu, Y. et al. Facet-dependent acidic and catalytic properties of sulfated titania solid superacids. *Chem. Commun.* **51,** 14219–14222 (2015).

[2] Yang, X. H., Li, Z., Sun, C., Yang, H. G. & Li, C. Hydrothermal stability of {001} faceted anatase TiO2. *Chem. Mater.* **23,** 3486–3494 (2011).

[3] Rothwell, W. P., Shen, W. & Lunsford, J. H. Solid-state phosphorus-31 NMR of a chemisorbed phosphonium ion in HY zeolite: observation of proton-phosphorus-31 coupling in the solid-state. *J. Am. Chem. Soc.* **106,** 2452–2453 (1984).

[4] Zheng, A., Huang, S.-J., Liu, S.-B. & Deng, F. Acid properties of solid acid catalysts characterized by solid-state 31P NMR of adsorbed phosphorous probe molecules. *Phys. Chem. Chem. Phys.* **13,** 14889–14901 (2011).

[5] Kunat, M., Girol, S. G., Burghaus, U. & Wöll, C. The Interaction of water with the oxygen-terminated, polar surface of ZnO. *J. Phys. Chem. B* **107,** 14350−14356 (2003).

[6] Gordon, T. R. et al. Nonaqueous synthesis of TiO2 nanocrystals using TiF4 to engineer morphology, oxygen vacancy concentration, and photocatalytic activity. *J. Am. Chem. Soc.* **134,** 6751−6761 (2012).

[7] D'Arienzo, M. et al. Photogenerated defects in shape-controlled TiO2 anatase nanocrystals: a probe to evaluate the role of crystal facets in photocatalytic processes. *J. Am. Chem. Soc.* **133,** 17652–17661 (2011).

[8] Liu, G. et al. Enhanced photoactivity of oxygen-deficient anatase TiO2 sheets with dominant {001} facets. *J. Phys. Chem. C* **113,** 21784–21788 (2009).

[9] Pan, J., Liu, G., Lu, G. Q. & Cheng, H.-M. On the true photoreactivity order of {001}, {010}, and {101} facets of anatase TiO2 crystals. *Angew. Chem. Int. Ed.* **50,** 2133–2137 (2011).

[10] Wei, W., Yaru, N., Chunhua, L. & Zhongzi, X. Hydrogenation of TiO2 nanosheets with exposed {001} facets for enhanced photocatalytc activity. *RSC Adv.* **2,** 8286–8288 (2012).

[11] Zárate, R. A., Fuentes, S., Wiff, J. P., Fuenzalida, V. M. & Cabrera, A. L. Chemical composition and phase identification of sodium titanate nanostructures grown from titania by hydrothermal processing. *J. Phys. Chem. Solids* **68,** 628–637 (2007).

[12] Ohtani, B., Prieto-Mahaney, O. O., Li, D. & Abe, R. What is Degussa (Evonik) P25? crystalline composition analysis, reconstruction from isolated pure particles and photocatalytic activity test. *J. Photochem. Photobiol., A* **216,** 179–182 (2010).

[13] Jensen, H., Joensen K. D., Jørgensen, J.-E., Pedersen J. S. & Søgaard G. Characterization of nanosized partly crystalline photocatalysts. *J. Nanoparticle Res.* **6,** 519–526 (2004).

[14] Simonsen, M. E., Jensen, H., Li, Z. & Sogaard, E. G. Surface properties and photocatalytic activity of nanocrystalline titania films. *J. Photochem. Photobiol., A* **200,** 192–200 (2008).

[15] Zhang, K. et al. An order/disorder/water junction system for highly efficient co-catalyst-free photocatalytic hydrogen generation. *Energy Environ. Sci.* **9,** 499–503 (2016).

[16] Kao, H.-M., Yu, C.-Y. & Yeh, M.-C. Detection of the inhomogeneity of brønsted acidity in H-mordenite and H-β zeolites: a comparative NMR study using trimethylphosphine and trimethylphosphine oxide as 31P NMR probes. *Micropor. Mesoporo. Mater.* **53,** 1–12 (2002).

[17] Zhao, Q. et al. Discernment and quantification of internal and external acid sites on zeolites H-ZSM-5. *J. Phys. Chem. B* **106,** 4462–4469 (2002).

[18] Luo, Q. et al. Using trimethylphosphine as a probe molecule to study the acid sites in Al−MCM-41 materials by solid-state NMR spectroscopy. *J. Phys. Chem. B* **107,** 2435–2442 (2003).

[19] Zhang, H. et al. Reactivity enhancement of 2-propanol photocatalysis on SO42−/TiO2: insights from solid-state NMR spectroscopy. *Environ. Sci. Technol.* **42,** 5316–5321 (2008).

[20] Kreissl, H. T. et al. Niobium oxides: correlation of acidity with structure and catalytic performance in sucrose conversion to 5-hydroxymethylfurfural. *J. Catal.* **338,** 329–339 (2016).

[21] Peng, Y.-K. et al. Trimethylphosphine-assisted surface fingerprinting of metal oxide nanoparticle by 31P solid-state NMR: a zinc oxide case study. *J. Am. Chem. Soc.* **138,** 2225–2234 (2016).

[22] Vanderbilt, D. Soft self-consistent pseudopotentials in a generalized eigenvalue formalism. *Phys. Rev. B: Condens. Matter* **41,** 7892–7895 (1990).

[23] Payne, M. C., Teter, M. P., Allan, D. C., Arias, T. A. & Joannopoulos, J. D. Iterative minimization techniques for ab initio total-energy calculations: molecular dynamics and conjugate gradients. *Rev. Mod. Phys.* **64,** 1045–1097 (1992).

[24] Perdew, J. P. et al. Atoms, molecules, solids, and surfaces: Applications of the generalized gradient approximation for exchange and correlation. *Phys. Rev. B: Condens. Matter* **46,** 6671–6687 (1992).

[25] Hanaor, D. A. H. & Sorrell, C. C. Review of the anatase to rutile phase transformation. *J. Mater. Sci.* **46,** 855–874 (2011).
